# Accuracy Evaluation and Comparison of 14 Diagnostic Markers for Nasopharyngeal Carcinoma: A Meta-Analysis

**DOI:** 10.3389/fonc.2020.01779

**Published:** 2020-09-18

**Authors:** Yiwei Feng, Wei Xia, Guangyao He, Rongdan Ke, Lei Liu, Mao Xie, Anzhou Tang, Xiang Yi

**Affiliations:** ^1^Department of Otolaryngology-Head & Neck Surgery, First Affiliated Hospital of Guangxi Medical University, Nanning, China; ^2^First Clinical Medical College, Guangxi Medical University, Nanning, China; ^3^Key Laboratory of Early Prevention and Treatment for Regional High Frequency Tumor, Nanning, China

**Keywords:** nasopharyngeal carcinoma, EB virus, diagnostic, screening, meta-analysis

## Abstract

The aim of the present study was to collect published studies and compare the diagnostic accuracy of different markers for nasopharyngeal carcinoma (NPC). We systematically searched PubMed/MEDLINE, EMBASE, Cochrane Library, CNKI, and Wanfang for relevant studies until April 29, 2020. The revised Quality Assessment of Diagnostic Accuracy Studies (QUADAS-2) tool was used to evaluate the methodological quality of the studies. The sensitivity, specificity, positive likelihood ratio (PLR), negative likelihood ratio (NLR), diagnostic odds ratio (DOR), and area under the curve (AUC) values of the diagnostic markers were combined by a bivariate mixed effect model to compare their diagnostic accuracy. We explored heterogeneity through meta-regression. In total, 244 records from 101 articles were included, with 49,432 total study subjects (13,109 cases and 36,323 controls). EA-IgG, Zta-IgG, and Epstein–Barr virus (EBV) DNA load in non-invasive nasopharyngeal brushings (EBV-DNA brushings) have both high sensitivity and specificity, EBNA1-IgG and VCA-IgG have only high sensitivity, and EBNA1-IgA, VCA-IgA, Rta-IgG, Zta-IgA, HSP70, and serum sialic acid (SA) have only high specificity. The bivariate mixed effect model of EA-IgA had a significant threshold effect. Meta-regression analysis showed that ethnicity affected EBNA1-IgA, EBNA1-IgG, VCA-IgA, and EBV DNA load in plasma, test methods affected EBNA1-IgG, publication year affected VCA-IgA, and sample size affected Rta-IgG. There was significant publication bias for VCA-IgA and Rta-IgG (*P* < 0.05). EA-IgG, Zta-IgG, and EBV-DNA brushings are good diagnostic markers for NPC. The diagnostic accuracy was influenced by publication year, sample size, test methods, and ethnicity.

## Introduction

Nasopharyngeal carcinoma (NPC) is an epithelial carcinoma originating from the inner layer of the mucous membrane on the superior and lateral of the nasopharynx. Compared with other malignant tumors, the incidence rate of NPC is relatively low, but its global distribution is extremely uneven (1); more than 70% of new cases are concentrated in East and Southeast Asia (2). Because of its small primary foci, NPC has no typical clinical symptoms in the early stages (stages I and II), and the tumor easily invades adjacent tissues and organs, resulting in complex and diverse clinical symptoms; thus, most patients have already reached advanced stages (stages III and IV) when they are diagnosed (3, 4). Therefore, the screening and early diagnosis of NPC are very important.

Epstein–Barr virus (EBV) is generally considered to be one of the risk factors for NPC (5–7). EBV-related antibodies have received extensive attention as diagnostic markers for NPC, and most of them maintain increased levels for an average of 38 months at the preclinical stage (3, 8, 9), which has high diagnostic predictive value. Moreover, measuring EBV-related antibodies has the characteristics of simple sample acquisition, rapid testing, convenience, and low cost (10, 11); thus, it is widely used in the screening of NPC in endemic areas. With the development of quantitative PCR (qPCR), detecting EBV-DNA load in serum, plasma, blood cells, and nasopharyngeal exfoliated cells has gradually become one of the common diagnostic methods for NPC (12–14). At the same time, some serum tumor markers, such as heat shock protein 70 (HSP70) and sialic acid (SA), play important roles in the development of NPC (15–17). Their content in serum is closely related to the progression of NPC, which makes these markers potential diagnostic markers of NPC, and more researchers have focused on this field.

Many studies have evaluated EBV-related antibodies, EBV-DNA load, and some tumor markers in the clinical diagnosis of NPC patients. However, due to different ethnicities, sample sizes, test methods, and other factors, these studies have obtained different sensitivities and specificities. To systematically evaluate the diagnostic efficacy of these markers and compare them with each other to find markers suitable for large-scale population screening and early clinical diagnosis, we conducted this meta-analysis.

## Methods

### Meta-Analysis

We conducted this meta-analysis according to the Preferred Reporting Items for Systematic Reviews and Meta-Analyses (PRISMA) and the guidance of the Cochrane Handbook for Systematic Reviews of Interventions.

### Search Strategy

We systematically searched PubMed/MEDLINE, EMBASE, Cochrane Library, CNKI, and Wanfang for all relevant studies up to April 29, 2020. The search language was restricted to Chinese and English, and the key words used for the search terms included the following: (“nasopharyngeal carcinoma” OR “Carcinoma, Nasopharyngeal” OR “Carcinomas, Nasopharyngeal”), (“VCA” OR “EBNA” OR “EA” OR “Zta” OR “BZLF1” OR “Rta” OR “BELF1” OR “HSP70” OR “Serum sialic acid” OR “EBV DNA”), and (“blood” OR “serum” OR “plasma” OR “blood cell” OR “leukocyte” OR “lymphocyte” OR “brush” OR “brushings”). In addition, the reference lists of relevant studies were manually searched for potential eligible studies. The search strategy is presented in Supplementary Materials.

### Study Selection and Data Extraction

To find suitable diagnostic markers for large-scale population screening and early clinical diagnosis while avoiding bias due to different experimental designs, we used the following inclusion criteria: (1) retrospective studies on the evaluation of diagnostic markers in NPC, (2) NPC was confirmed by pathological examination, (3) the control group should be healthy individuals or non-NPC patients confirmed by pathological examination, (4) the samples were peripheral blood serum, plasma, blood cells, or non-invasive nasopharyngeal brushings, and (5) the articles included sufficient data to construct a 2 × 2 table, including true positive (TP), false positive (FP), false negative (FN), and true negative (TN) counts. The exclusion criteria were as follows: (1) irrelevant topics, (2) letters, comments, editorials, conference abstracts, reviews, guidelines, and case reports, and (3) non-clinical studies, such as animal or cell experiments. Two investigators independently completed the study selection and data extraction. Any disagreements were resolved by a third investigator.

### Quality Assessment

Based on the recommendations of the Cochrane Collaboration, we used the revised Quality Assessment for Studies of Diagnostic Accuracy (QUADAS-2) tool to evaluate the quality of each included study. This tool assessed the bias risk and clinical applicability of the studies based on four key areas: “patient selection,” “index test,” “reference standard,” and “flow and timing.” Three investigators independently used the quality assessment tool and flowchart to evaluate the studies, and any differences were resolved by consensus.

### Statistical Analysis

We performed statistical analysis and analyzed each NPC diagnostic marker separately and then compared their sensitivity, specificity, and area under the curve (AUC) values. Specifically, first, the Pearson coefficient was used to evaluate the threshold effect, and then a bivariate mixed effect model was used. We calculated the following parameters and their 95% confidence intervals (CIs): sensitivity, specificity, positive likelihood ratio (PLR), negative likelihood ratio (NLR), diagnostic odds ratio (DOR), and AUC values of the summary receiver operating characteristic (SROC) curve. Then, a forest plot of sensitivity and specificity was drawn. We examined the forest plot and combined the *Q* test and *I*^2^ statistic to check the heterogeneity within studies. *P* < 0.05 and *I*^2^ > 50% indicated significant heterogeneity. Fagan's plot was used to test the relationship between the pre-test probability and post-test probability. Sensitivity analysis consisted of the following experiments: a graphical depiction of residual-based goodness-of-fit and a chi-squared probability plot of squared Mahalanobis distances were used for the assessment of the bivariate normality assumption, a spike plot was used to check observations that affect Cook's distance, and outliers of standardized predicted random effects were checked by scatter plots. Finally, publication bias was checked through Deek's funnel plot. *P* < 0.05 was considered significant. The statistical analyses were performed using Stata version 16.0 (Stata Corp, College Station, Texas, USA) and Review Manager 5.3 (The Nordic Cochrane Center, Copenhagen).

## Results

### Study Selection and General Characteristics

**Figure 1:**
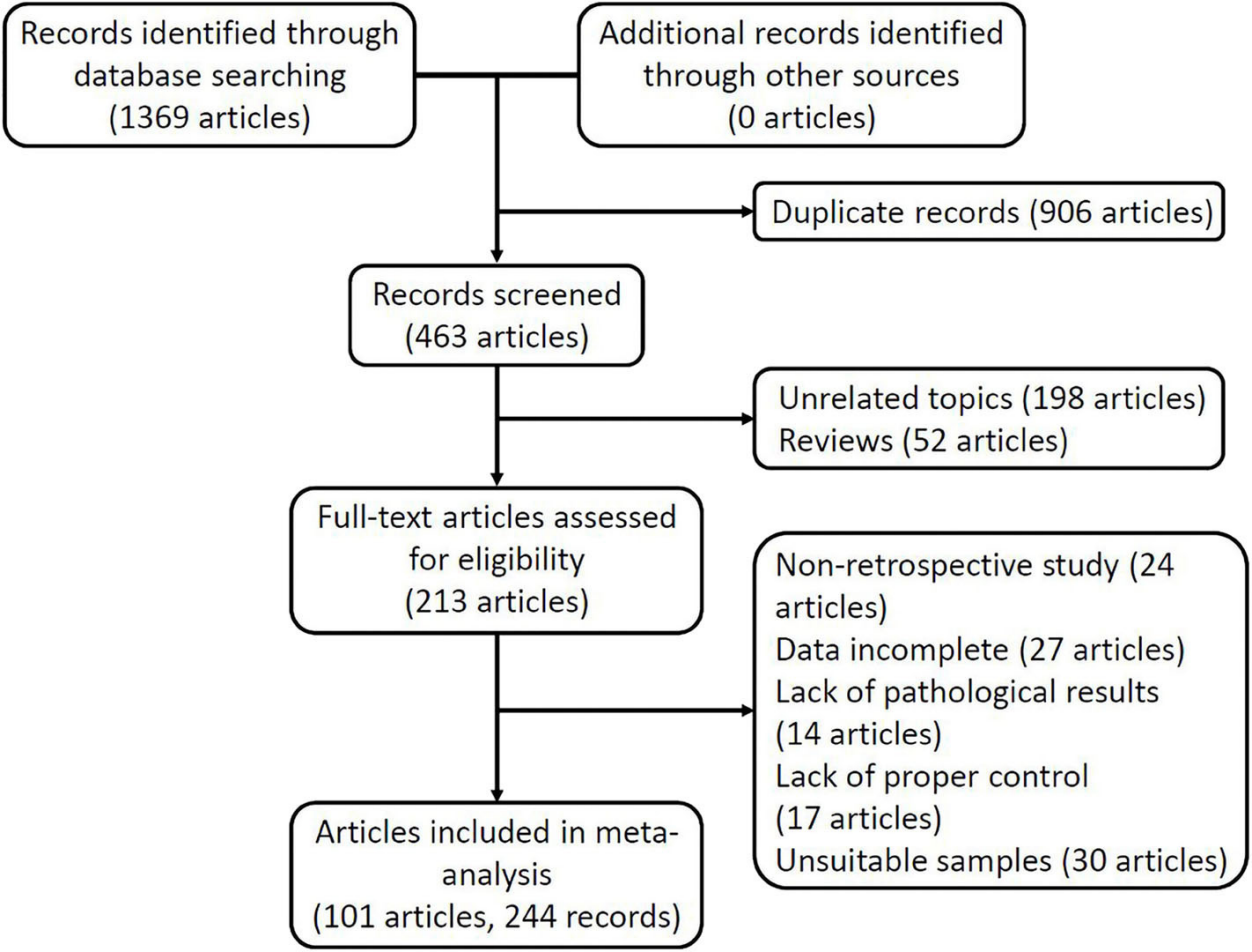
PRISMA flow diagram of the study selection process.

Figure 1 shows the flow of the study selection and data extraction processes. Based on the search strategy, we obtained 1,369 articles from five online electronic databases. After excluding 906 articles with duplicate data and publications, the titles and abstracts of the remaining 463 articles were checked, and 198 articles unrelated to the subject of the study and 52 review articles were excluded. We downloaded the full text of 213 articles for further review and excluded 24 non-retrospective articles, 27 articles that could not provide enough information to construct a complete 2 × 2 table, 14 articles that lacked pathological examination, 17 articles that lacked an appropriate control group, and 30 articles that did not meet the requirements of the sample. Finally, 244 records from 101 articles remained for meta-analysis, with a total sample size of 49,432 study subjects (13,109 cases and 36,323 controls). These studies were published from 1994 to 2019.

### Quality Assessment

QUADAS-2 was used to evaluate the methodological quality of the included study, and the detailed quality evaluation form is provided in Supplementary Materials. The results showed that the bias risk mainly came from patient selection. Some studies did not explain whether patients were included in a continuous or random way, for which only “unclear” or “high risk” was given. Some studies did not include all cases. Additionally, a small number of studies did not set a threshold for testing in advance. Overall, the quality of the included studies was high.

### Pooled Results

**Figure 2:**
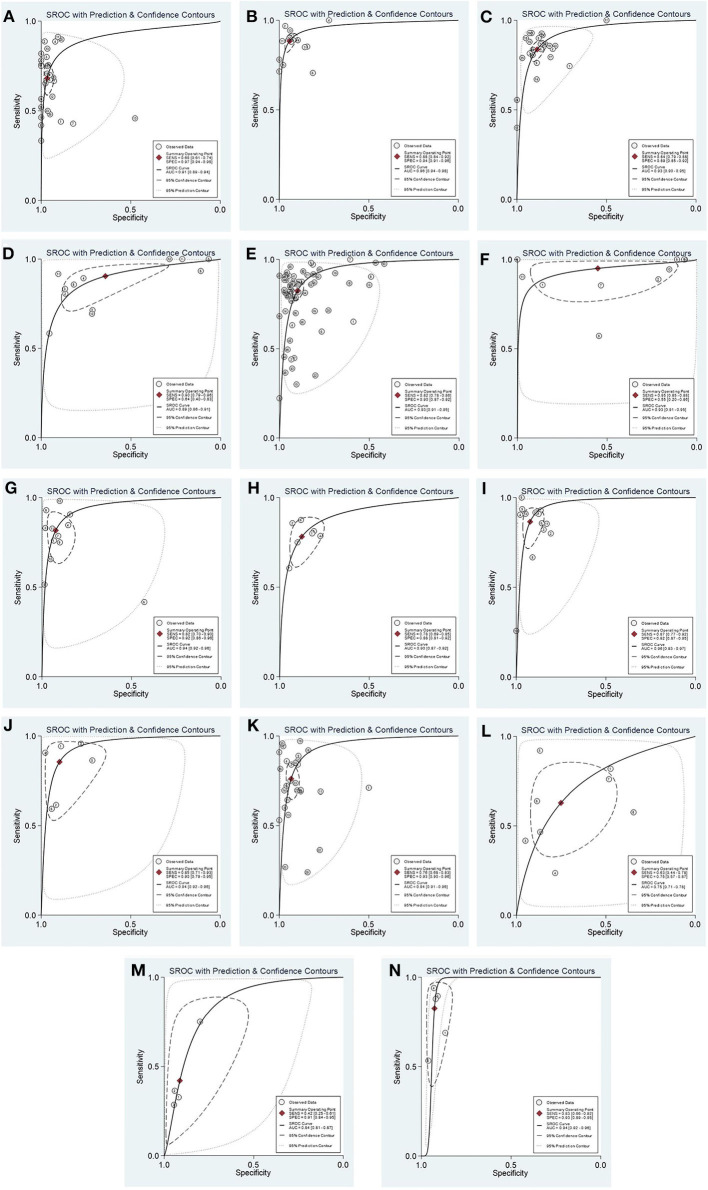
The SROC curves of each diagnostic marker for NPC. EA-IgA **(A)**; EA-IgG **(B)**; EBNA1-IgA **(C)**; EBNA1-IgG **(D)**; VCA-IgA **(E)**; VCA-IgG **(F)**; Rta-IgG **(G)**; Zta-IgA **(H)**; Zta-IgG **(I)**; EBV-DNA brushings **(J)**; EBV-DNA plasma **(K)**; EBV-DNA PMB **(L)**; HSP70 **(M)**; SA **(N)**. The curve of EA-IgA **(A)** showed a significant “shoulder arm” shape, suggesting that there may be a significant threshold effect between the included studies.

Table 1 shows the combined results of the sensitivity, specificity, PLR, NLR, DOR, AUC values, pre-test probability, and post-test probability of each diagnostic marker. Generally, when PLR is >10 and NLR is <0.1, the diagnostic marker can obtain high efficiency, and accordingly, the DOR and post-test probability will increase. It should be noted that in the effect model of EA-IgA, the threshold effect test *P* < 0.001, and the correlation was −0.02 after the SROC curve was fit, and the data points in the figure were checked, showing a significant “shoulder arm” shape (Figure 2A), which suggests that there may be a significant threshold effect between the included studies. When there is a significant threshold effect in the effect model, the combined results between the individual effect quantities become unstable, and more attention should be paid to the SROC curves and AUC values. No significant threshold effect was found in the effect models of the other diagnostic markers. If we define ≥85% as a high level and <75% as a low level, then the combined sensitivity and specificity of EA-IgG, Zta-IgG, and EBV-DNA brushings were at a high level, EBNA1-IgG and VCA-IgG had only combined sensitivity at a high level, EBNA1-IgA, VCA-IgA, Rta-IgG, Zta-IgA, HSP70, and SA had only combined specificity at a high level, and EBV-DNA peripheral blood cells (PMB) had both combined sensitivity and specificity at a low level. Combined with other effects, EA-IgG, EA-IgG, and EBV-DNA brushings are good diagnostic markers for NPC. The SROC curves of the diagnostic markers are listed in Figure 2 for reference, and other plots not listed can be found in Supplementary Materials.

**Table 1:** Combined results of sensitivity, specificity, PLR, NLR, DOR, AUC values, pre-test probability, and post-test probability of each diagnostic marker.

**Diagnostic**	**Included**	**Record**	**Sensitivity**	**Specificity**	**PLR**	**NLR**	**DOR**	**AUC**	**Pre-test**	**Post-test**
**marker**	**studies**	**number**	**(95% CI)**	**(95% CI)**	**(95% CI)**	**(95% CI)**	**(95% CI)**	**(95% CI)**	**probability (%)**	**probability (%)**
EA-IgA	(10, 18–43)	29	0.68 (0.61–0.74)	0.97 (0.94–0.98)	20.5 (11.4–36.7)	0.33 (0.27–0.41)	62 (32–121)	0.91 (0.94–0.94)	8	84
EA-IgG	(24, 25, 27–29, 31, 32, 38, 44–49)	15	0.88 (0.84–0.92)	0.94 (0.91–0.96)	15.4 (10.4–23.0)	0.12 (0.09–0.17)	125 (84–187)	0.89 (0.86–0.91)	3	79
EBNA1-IgA	(8, 10, 19, 21, 27, 31, 32, 37, 44, 48, 50–63)	26	0.84 (0.79–0.88)	0.89 (0.85–0.92)	7.4 (5.7–9.7)	0.18 (0.14–0.24)	41 (29–56)	0.93 (0.90–0.95)	4	65
EBNA1-IgG	(10, 24, 31, 49, 51–54, 56–58, 64)	12	0.90 (0.79–0.96)	0.64 (0.40–0.83)	2.5 (1.4–4.6)	0.15 (0.08–0.28)	17 (8–36)	0.89 (0.86–0.91)	4	39
VCA-IgA	(8, 10, 19–40, 42–45, 47, 49–51, 54, 57, 59, 60, 62–88)	68	0.82 (0.78–0.86)	0.90 (0.87–0.92)	8.2 (6.6–10.3)	0.19 (0.16–0.24)	42 (31–57)	0.93 (0.91–0.95)	5	67
VCA-IgG	(24, 25, 27, 29, 31, 49, 54, 57, 77, 87, 89)	11	0.95 (0.85–0.98)	0.55 (0.20–0.86)	2.1 (0.9–5.0)	0.09 (0.03–0.31)	23 (4–141)	0.93 (0.91–0.95)	2	35
Rta-IgG	(10, 18, 26, 37, 40, 43, 44, 77, 82, 85, 90, 91)	12	0.82 (0.70–0.90)	0.92 (0.86–0.96)	10.6 (5.5–20.8)	0.20 (0.11–0.34)	54 (19–151)	0.94 (0.92–0.96)	5	73
Zta-IgA	(10, 19, 52, 54, 56, 62, 63)	7	0.78 (0.69–0.85)	0.88 (0.81–0.92)	6.3 (4.2–9.4)	0.25 (0.18–0.35)	25 (15–43)	0.90 (0.87–0.92)	6	61
Zta-IgG	(24, 51–56, 58, 77, 92–95))	13	0.87 (0.77–0.92)	0.92 (0.87–0.95)	11.2 (6.8–18.3)	0.15 (0.08–0.25)	77 (36–162)	0.96 (0.93–0.97)	4	74
EBV-DNA brushings	(59, 78, 96–99)	6	0.85 (0.71–0.93)	0.90 (0.79–0.95)	8.3 (4.0–17.4)	0.16 (0.08–0.34)	51 (18–146)	0.94 (0.92–0.96)	4	68
EBV-DNA plasma	(13, 19, 34, 36, 39, 58, 59, 62, 68–70, 76, 79, 82–84, 87, 100–109)	28	0.76 (0.68–0.83)	0.93 (0.90–0.96)	11.4 (7.4–17.6)	0.26 (0.19–0.36)	45 (23–85)	0.94 (0.91–0.96)	6	76
EBV-DNA PMB	(68, 103, 104, 106, 109–112)	8	0.63 (0.44–0.78)	0.75 (0.57–0.87)	2.5 (1.3–4.8)	0.49 (0.31–0.80)	5 (2–14)	0.75 (0.7–0.78)	11	39
HSP70	(80, 81, 113)	4	0.42 (0.25–0.61)	0.91 (0.84–0.95)	4.8 (3.0–7.7)	0.64 (0.48–0.84)	8 (4–14)	0.84 (0.81–0.87)	14	55
SA	(18, 67, 72, 114, 115)	5	0.83 (0.66–0.92)	0.93 (0.89–0.95)	11.3 (7.6–16.8)	0.19 (0.09–0.39)	60 (24–148)	0.94 (0.92–0.96)	4	74

PLR, positive likelihood ratio; NLR, negative likelihood ratio; DOR, diagnostic odds ratio; AUC, area under the curve.

*The effect model of EA-IgA has a significant threshold effect, the combined results between the individual effect quantities become unstable, and more attention should be paid to the SROC curves and AUC values*.

### Heterogeneity, Sensitivity Analysis, and Publication Bias

**Figure 3:**
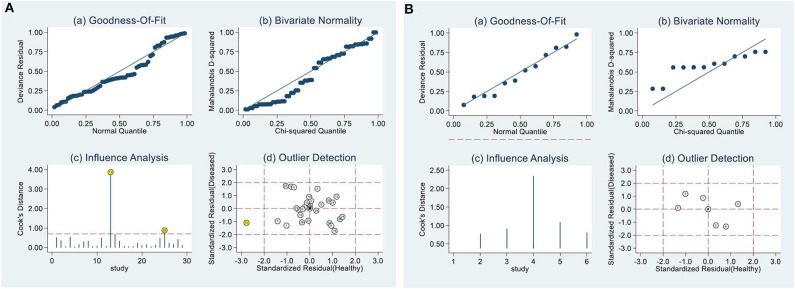
Sensitivity analysis. **(A)** Result of EA-IgG, **(B)** EBV-DNA brushings. **(A)** and **(B)** are composed of 4 parts, goodness-of-fit **(a)**, bivariate normality **(b)**, influence analysis **(c)** and outlier detection **(d)** show that only a few studies are beyond the scope of the reference line, indicating that the effect model is stable.

Because only the combined specificity in SA and combined sensitivity in EA-IgG and EA-IgA showed low heterogeneity, we explored the potential sources of heterogeneity. When the number of articles included in a meta-analysis is <10, meta-regression analysis is not recommended in the Cochrane Handbook for Systematic Reviews of Interventions. Additionally, EA-IgA had a significant threshold effect, so we only analyzed the sensitivity (Figure 3) and publication bias of EA-IgA, EBV-DNA brushings, EBV-DNA PMB, HSP70, and SA, whereas other diagnostic markers were additionally analyzed by meta-regression analysis. The goodness-of-fit and bivariate normality of the sensitivity analysis show the studies along the reference line, whereas the influence analysis and outlier detection show which studies have a significant impact on the effect model. Only a few studies were beyond the scope of the reference line, and the results after removing these studies one by one did not change significantly. This indicates that the effect model is stable, and few studies affect the pooling results. The meta-regression analysis mainly included the year of publication, sample size, test method, and ethnicity. The results are shown in Table 2. We found that ethnicity (Asian vs. non-Asian) has an effect on EBNA1-IgA, EBNA1-IgG, VCA-IgA, and EBV-DNA plasma, and the test method [ELISA vs. indirect fluorescent antibody (IFA)] affects EBNA1-IgG. Publication year (≥median vs. < median) affects VCA-IgA, whereas sample size (≥median vs. < median) affects Rta-IgG. The asymmetry in Deek's funnel plot showed that there was significant publication bias for VCA-IgA and Rta-IgG (*P* < 0.05), but no significant publication bias was found in the other effect models.

**Table 2:** Meta-regression analyses of potential sources of heterogeneity.

**Diagnostic marker**	**Variable**	**Coefficient**	***P*-value**	***I*^**2**^**
EA-IgG	Publication year	3.98	0.14	50
	Sample size	4.27	0.12	53
	Test method	3.22	0.20	38
	Ethnic	0.76	0.68	0
EBNA1-IgA	Publication year	0.12	0.94	0
	Sample size	3.27	0.20	39
	Ethnic	7.30	0.03^*^	73
EBNA1-IgG	Publication year	0.55	0.76	0
	Sample size	0.74	0.69	0
	Test method	6.50	0.04^*^	69
	Ethnic	8.87	0.01^*^	77
VCA-IgA	Publication year	6.37	0.04^*^	69
	Sample size	2.63	0.27	24
	Test method	3.41	0.18	41
	Ethnic	0.84	0.01^*^	76
VCA-IgG	Publication year	0.79	0.67	0
	Sample size	0.06	0.97	0
	Test method	0.54	0.76	0
	Ethnic	2.92	0.23	31
Rta-IgG	Publication year	1.44	0.49	0
	Sample size	6.97	0.03^*^	71
	Ethnic	1.58	0.45	0
Zta-IgG	Publication year	1.89	0.39	0
	Sample size	0.85	0.65	0
	Ethnic	1.84	0.40	0
EBV-DNA plasma	Publication year	5.39	0.07	63
	Sample size	2.41	0.30	17
	Ethnic	6.25	0.04^*^	68

* *P < 0.05 indicates a significant difference*.

## Discussion

By meta-analysis of published articles, we compared the diagnostic efficacy of 14 diagnostic markers for NPC. VCA-IgG had the highest combined sensitivity, EA-IgG had the highest combined specificity, and Zta-IgG had the highest combined AUC values. In general, the combined sensitivity, specificity, and diagnostic efficacy of EA-IgG, Zta-IgG, and EBV-DNA brushings are at a high level, and their sample acquisition methods are relatively simple. Additionally, the detection process is fast and inexpensive, which has high practical value in large-scale population screening and early clinical diagnosis.

At present, antibodies against EBV mainly include early antigen (EA), nuclear antigen 1 (EBNA1), viral capsid antigen (VCA), Rta protein encoded by the EBV immediate early gene BRLF1, and Zta protein encoded by the BZLF1 gene. When EBV is in incubation period, only EBNA1 can induce a strong antibody response (50). After entering the lysis cycle, Rta and Zta are encoded first, followed by EA. VCA is expressed only at the end of the EBV proliferation cycle (116), and the expression of these proteins will cause a strong antibody response in patients (10). In the preclinical stage, EBV replicates in the body of patients, and antibodies will remain at a high level, making these antibodies potential early diagnostic markers for NPC. Regarding the test method, compared with IFA, ELISA has the advantages of low cost and a standardized operation process (117, 118); moreover, the interpretation of the results is not affected by the subjective judgment of researchers, so it is easier to use in large-scale population screening.

The expression levels of different antibodies can indirectly reflect the replication of EBV, and measuring the EBV-DNA load provides a more intuitive reference. In 1999, Lo et al. (14) first proposed the use of qPCR to diagnose NPC by detecting the EBV-DNA load in plasma, and this method has good performance in disease development monitoring and prognosis prediction (119–121). Subsequently, related research on EBV-DNA load in blood cells and nasopharynx exfoliated cells was carried out successively and performed well. The BamH1-W sequence is a mature and reliable laboratory EBV-DNA detection method recommended by the WHO (122), and most primers are designed based on this sequence. With the commercialization and promotion of standardized test kits, the cost of learning and using this test method has been greatly reduced; thus, this test can be extensively applied in large-scale population screening and early clinical diagnosis.

During the occurrence and development of tumors, tumor cells express tumor antigens that are different from normal cells, and the immune system recognizes tumor antigens and produces autoantibodies (123, 124). SA is the acetylation product of neuraminic acid, an important component of cell membrane surface receptors. It has been indicated to be closely related to the occurrence, development, and metastasis of head and neck tumors (18, 125). The mechanism of abnormally increased SA may be that the glycoprotein and glycolipid on the cell membrane fall off and enter the blood circulation when the tumor cell structure is destroyed by immune cells (126). HSP70 is a highly effective inhibitor of apoptosis. Its basic expression level is very low, but it is highly expressed in a variety of tumors, such as NPC, breast cancer, and renal cell carcinoma (127, 128), and is considered an important marker of tumor occurrence and prognosis (129–131). The efficacy of serum tumor markers in the early diagnosis of NPC is attracting researchers' attention.

In addition, an increasing number of studies have pointed out that microRNAs, lncRNAs, and circRNAs are closely related to the physiological and pathological processes of NPC (132–134). Although these noncoding RNAs do not have the function of translating and coding proteins, they can inhibit the translation or degradation of target mRNAs through complete or incomplete complementary pairing, regulating downstream protein expression or signaling pathways; thus, they participate in the process of cell proliferation and differentiation. The abnormal expression of specific non-coding RNAs plays an important role in the occurrence and development of NPC and may become potential diagnostic markers.

In the screening and early diagnosis of NPC, it is important to reduce the missed diagnosis rate as much as possible and control the misdiagnosis rate within the acceptable range. Some prospective and retrospective studies have indicated that a combination of multiple diagnostic markers can achieve high sensitivity compared with using only a single diagnostic marker (10, 19–21, 44). When the patient obtains a positive result in screening or early diagnosis, further imaging and pathological examination and frequent follow-up visits can accurately determine whether the patient has NPC. Therefore, exploring the best combination of diagnostic markers will be the focus of future NPC screening and early diagnosis research.

There are some limitations in this study. First, the global distribution of NPC is uneven. Most of the articles we included were from Asia; thus, whether our results are applicable in non-NPC endemic areas needs to be further confirmed. Second, most of the case groups in the study included NPC patients in different stages, and the level of diagnostic markers among them may be different. Third, the control group was not entirely composed of healthy people and included some patients with non-NPC head and neck diseases, which may have a certain impact on the actual results. Fourth, other diseases caused by EBV (including infectious mononucleosis, Burkitt's lymphoma, etc.) may also lead to increased EBV-related antibodies, and the results of EBV-DNA load are FPs. Finally, our results showed heterogeneity. We conducted a meta-regression analysis of the publication year, sample size, test method, and ethnicity, but these items cannot fully explain the source of heterogeneity, and further research is needed.

## Data Availability Statement

All datasets presented in this study are included in the article/Supplementary Material.

## Author Contributions

YF and WX: conceptualization and validation. YF, WX, and GH: methodology and literature search. YF: software. YF and RK: writing—original draft preparation. LL, MX, and AT: writing—review and editing. XY: supervision. All authors have read and agreed to the published version of the manuscript.

## Conflict of Interest

The authors declare that the research was conducted in the absence of any commercial or financial relationships that could be construed as a potential conflict of interest.
