# Supplementary material for: Accuracy Evaluation and Comparison of 14 Diagnostic Markers for Nasopharyngeal Carcinoma: A Meta-Analysis
Source: Front Oncol. 2020 Sep 18;10:1779. doi: 10.3389/fonc.2020.01779 (PMC7531263; doi:10.3389/fonc.2020.01779)
Supplement: Supplementary file 1 [file Data_Sheet_1.docx]

Supplementary Material

**Methods S1: Search strategy**

# PUBMED (until April 29, 2020)

#
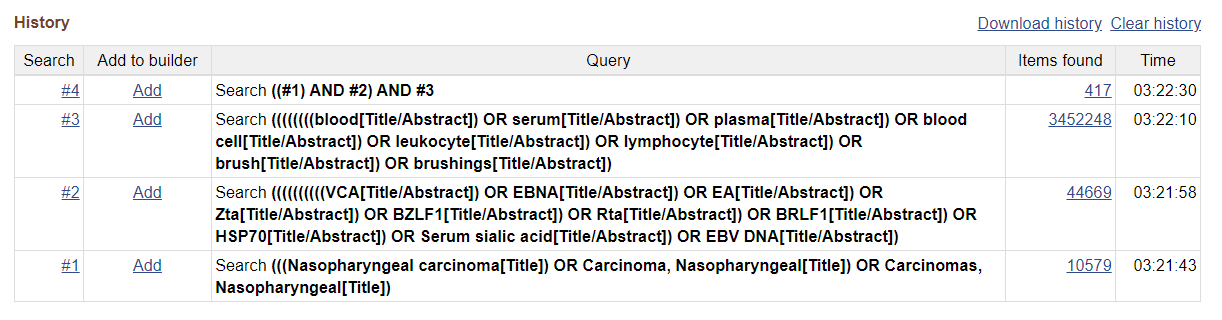


# Cochrane Library (until April 29, 2020)

# #1 MeSH descriptor: [Nasopharyngeal Carcinoma] explode all trees 109

# #2 (Nasopharyngeal carcinoma):ti,ab,kw 1246

# #3 #1 or #2 1246

# #4 (VCA):ti,ab,kw 43

# #5 (EBNA):ti,ab,kw 21

# #6 (EA):ti,ab,kw 2530

# #7 (ZTA):ti,ab,kw 2

# #8 (BZLF1):ti,ab,kw 0

# #9 (RTA):ti,ab,kw 91

# #10 (BRLF1):ti,ab,kw 0

# #11 (HSP70):ti,ab,kw 183

# #12 (Serum sialic acid):ti,ab,kw 36

# #13 (EBV DNA):ti,ab,kw 103

# #14 {OR #4-#13} 2986

# #15 (blood):ti,ab,kw 341898

# #16 (serum):ti,ab,kw 96889

# #17 (plasma):ti,ab,kw 95289

# #18 (blood cell):ti,ab,kw 38228

# #19 (leukocyte):ti,ab,kw 9858

# #20 (Lymphocyte):ti,ab,kw 15178

# #21 (brush):ti,ab,kw 1766

# #22 (brushings):ti,ab,kw 103

# #23 {OR #15-#22} 411765

# #24 #3 AND #14 AND #23 30

# Embase (until April 29, 2020)

#
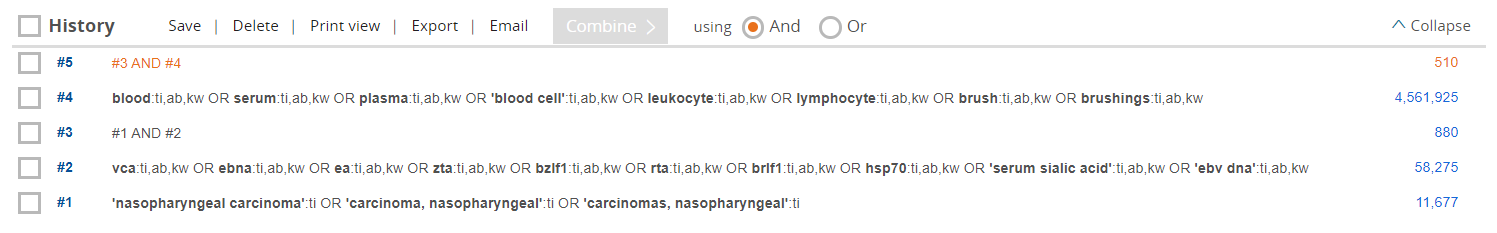


# CNKI (until April 29, 2020)

# ( ( (题名=‘鼻咽癌’ ) OR (title=‘鼻咽癌’ ) ) 并且 ( ( 摘要=VCA+EBNA1+EA+ZAT+BZLF1+Rta+BRLF1+HSP70+唾液酸+EBV DNA) OR ( abstract_en=VCA+EBNA1+EA+Zta+BZLF1+Rta+BRLF1+HSP70+Serum sialic acid+EBV DNA) ) ) 并且 ( 核心期刊=Y) (精确匹配)

# Results=223

# Wanfang (until April 29, 2020)

# 题目:“鼻咽癌”*（摘要:“VCA”+"EBNA1"+"EA"+“Zta”+“BZRF1”+"Rta"+"BRLF1"+"HSP70"+"唾液酸"+“Serum sialic acid”+“EBV DNA”）并且限定条件=北大核心

# Results=189

# (A). Sensitivity and specificity forest map of EA-IgA.

#
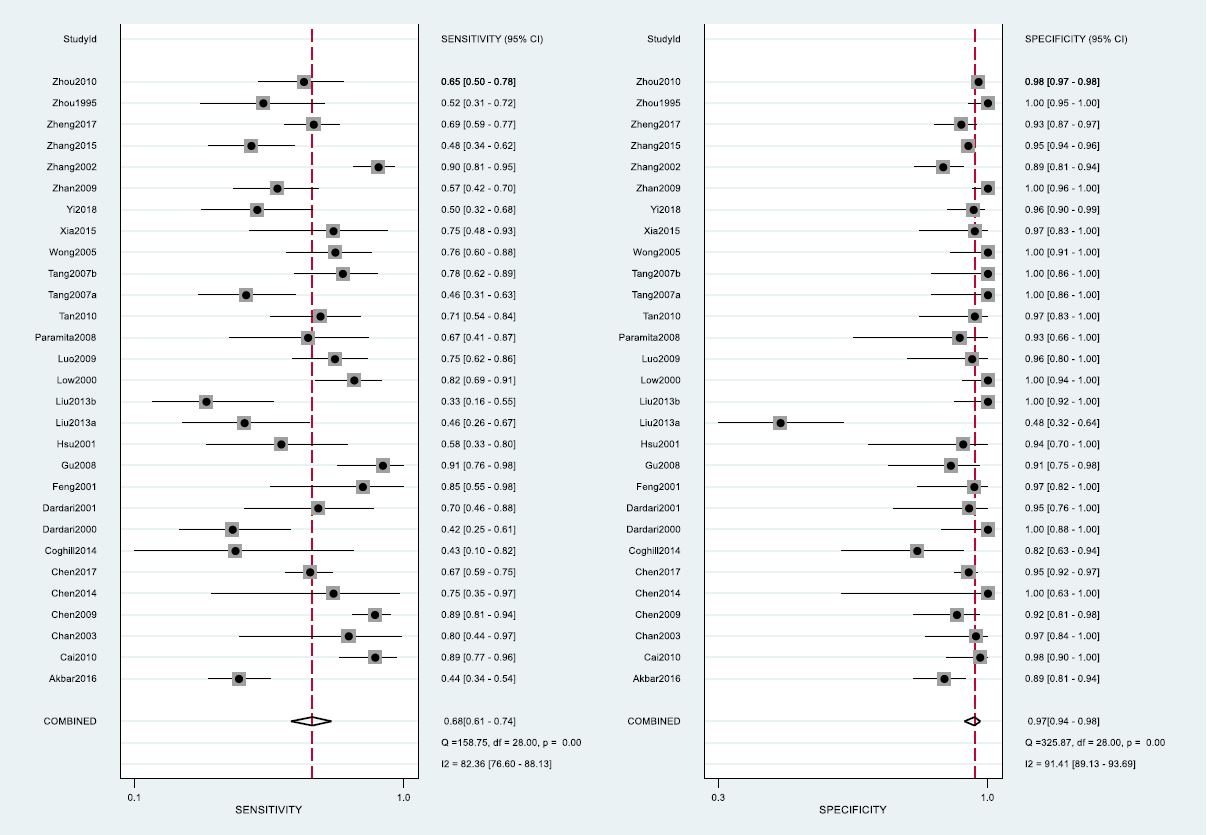


# (B). Fagan’s plot of EA-IgA.

#
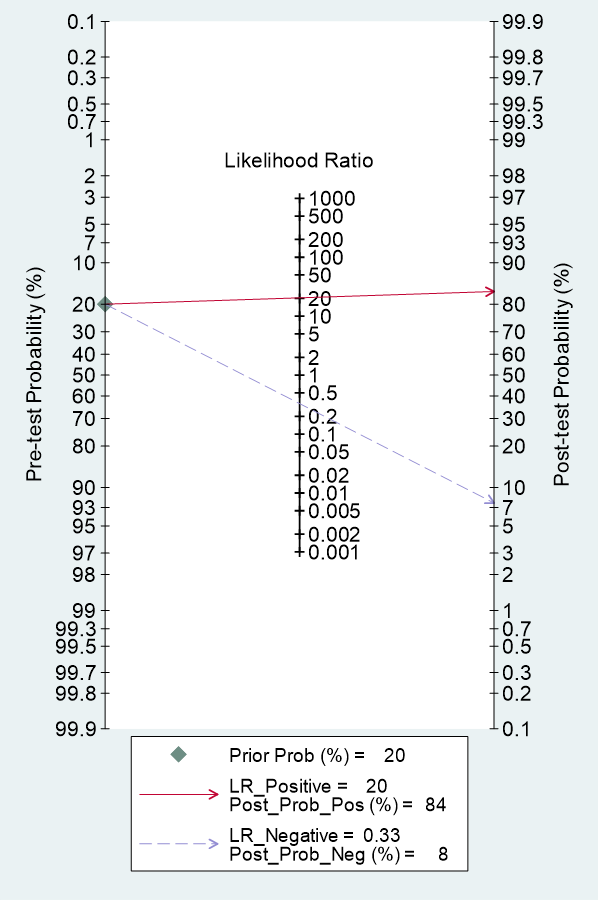


# (C). Sensitivity analysis of EA-IgA.

#
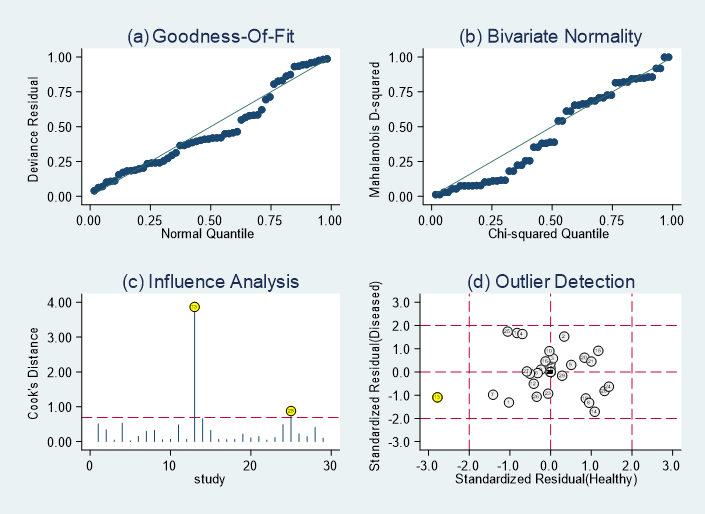


# (D). Deek’s funnel plot of EA-IgA.

#
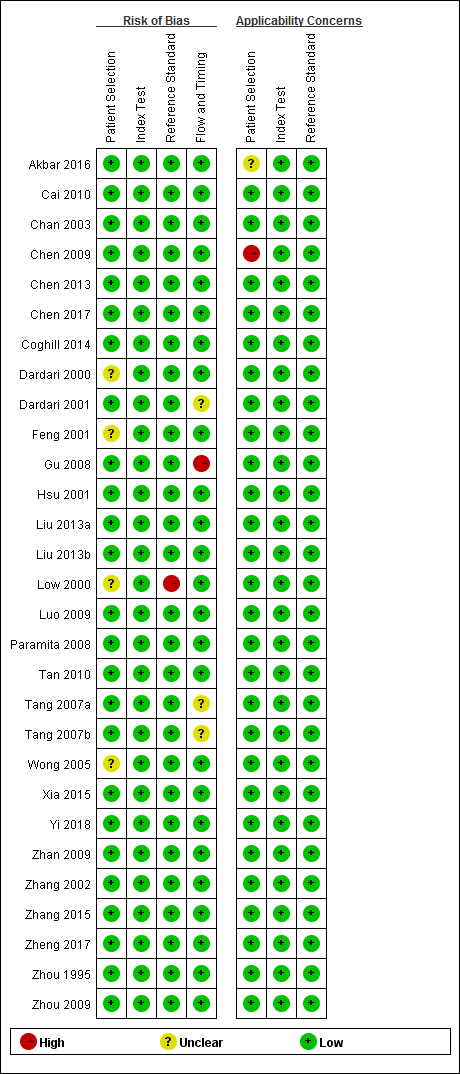

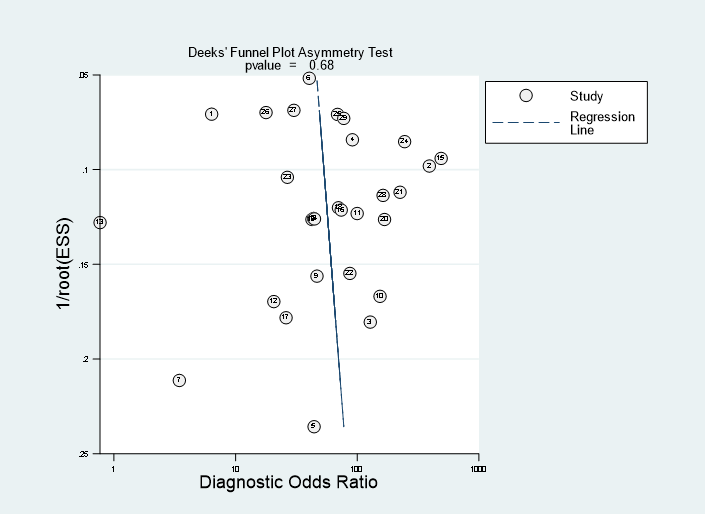


#
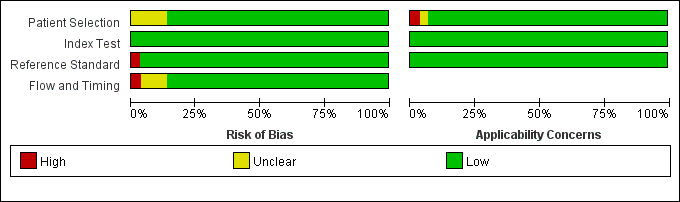
(E). Risk of bias summary of EA-IgA.

# Figure S1. Supplementary materials of EA-IgA. (A) Sensitivity and specificity forest map; (B) Fagan’s plot; (C) Sensitivity analysis; (D) Deek’s funnel plot; (E) Risk of bias summary.

# (A). Sensitivity and specificity forest map of EA-IgG.

#
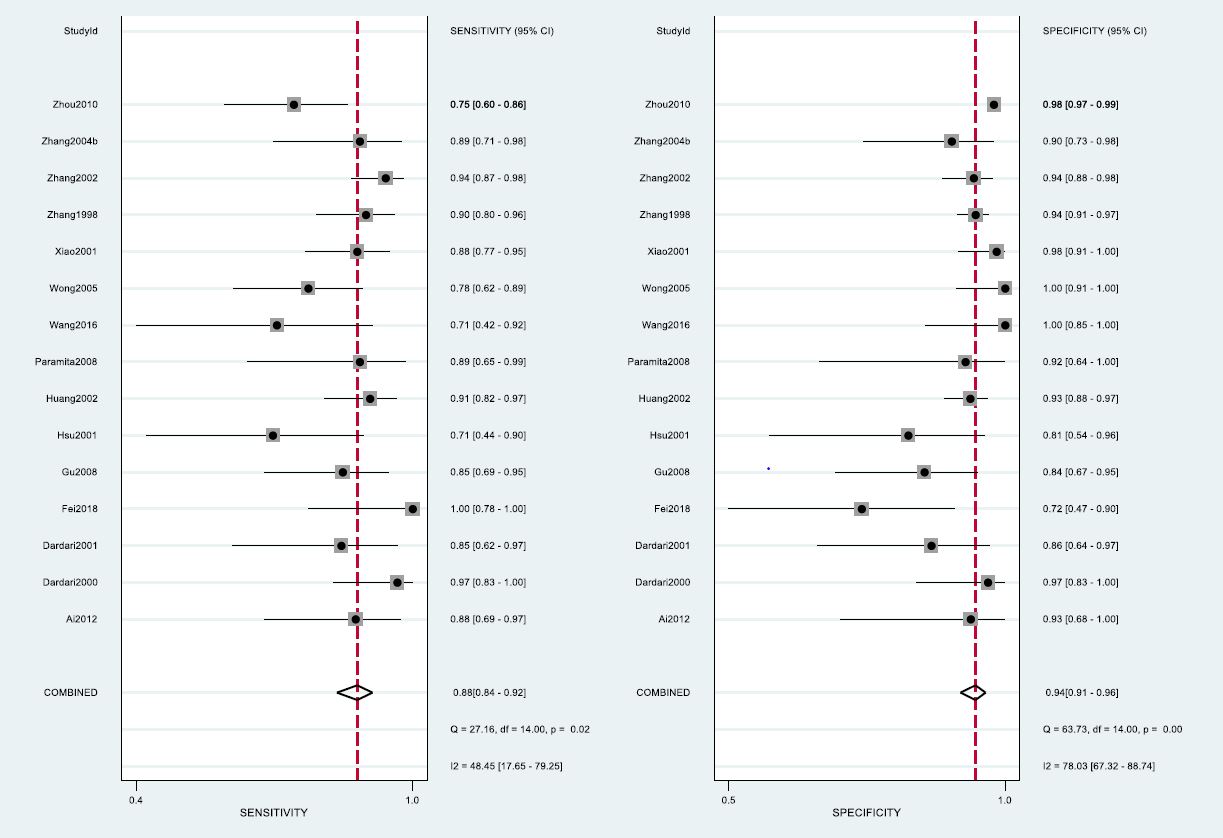


# (B). Fagan’s plot of EA-IgA.

#
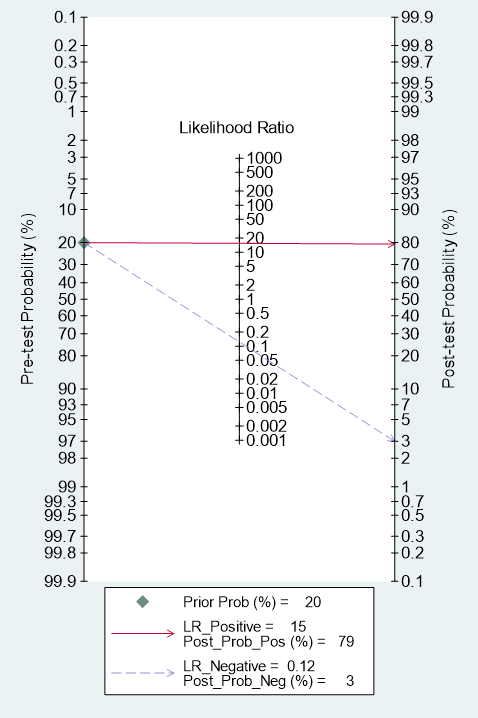


# (C). Deek’s funnel plot of EA-IgA.

#
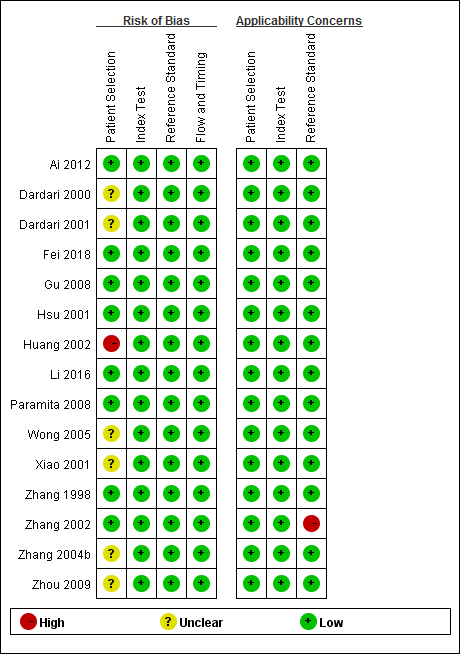

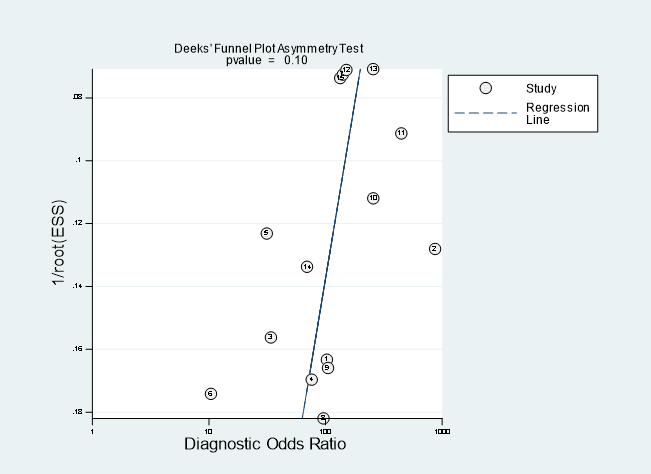


# (D). Risk of bias summary of EA-IgA.

#
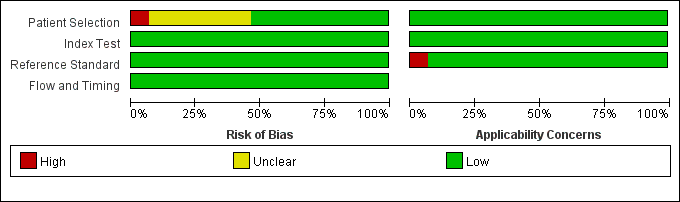


# Figure S2. Supplementary materials of EA-IgG. (A) Sensitivity and specificity forest map; (B) Fagan’s plot; (C) Deek’s funnel plot; (D) Risk of bias summary.

# (A). Sensitivity and specificity forest map of EBNA1-IgA.

#
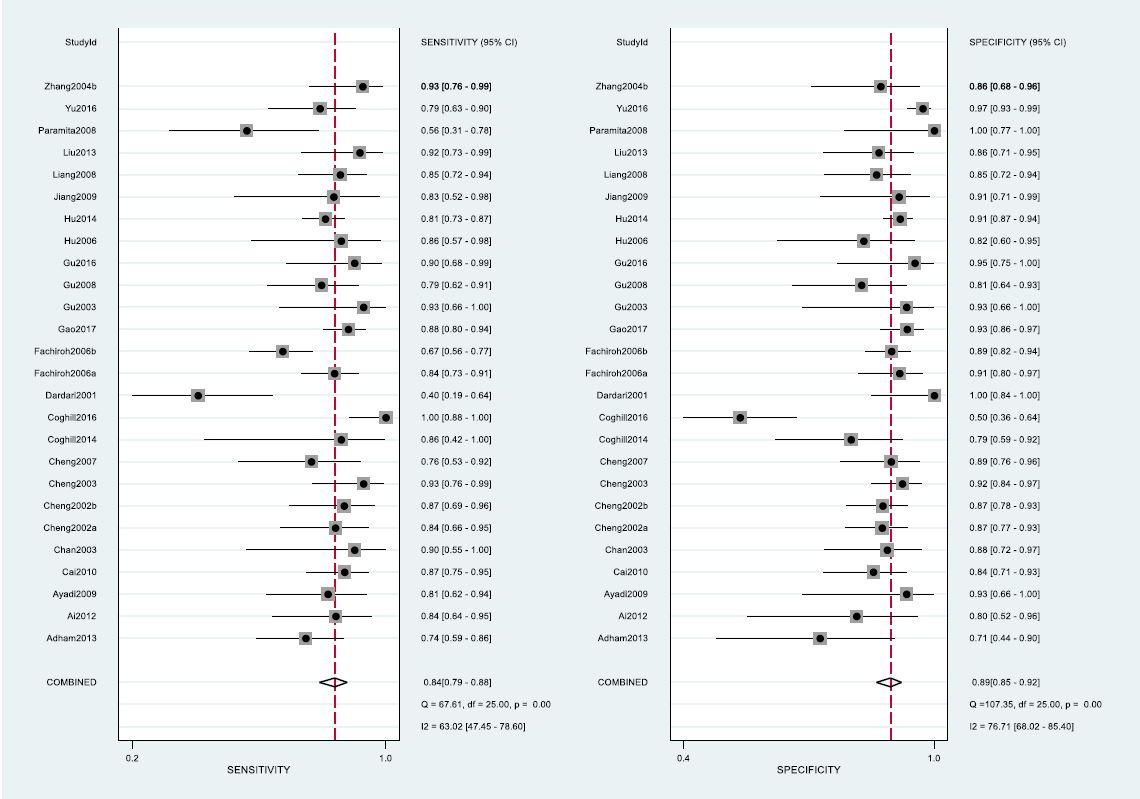


# (B). Fagan’s plot of EBNA1-IgA.

#
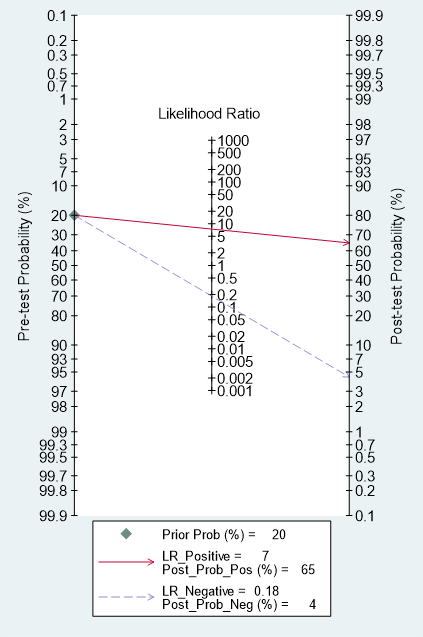


# (C). Sensitivity analysis of EBNA1-IgA.

#
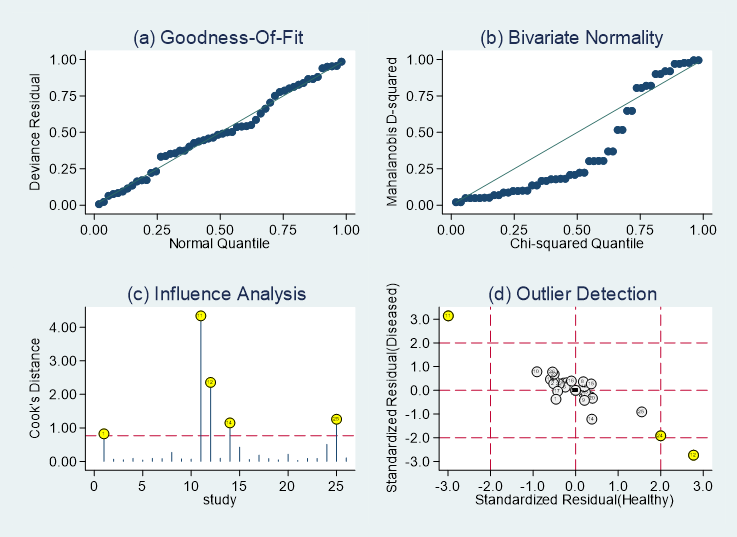


# (D). Deek’s funnel plot of EBNA1-IgA.

#
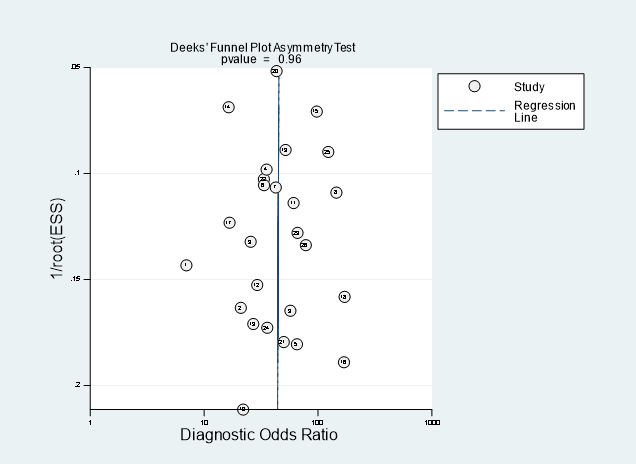


#
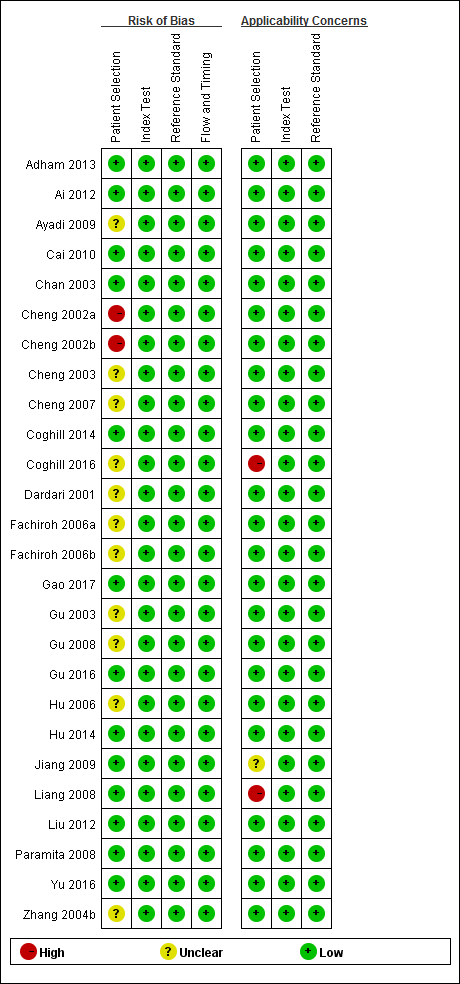


#
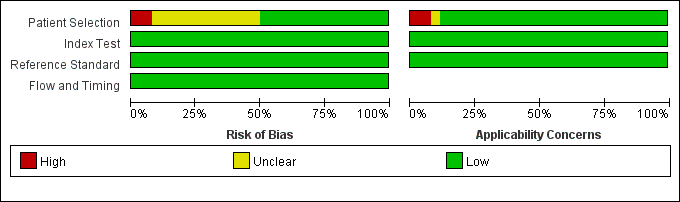
(E). Risk of bias summary of EBNA1-IgA.

# Figure S3. Supplementary materials of EBNA1-IgA. (A) Sensitivity and specificity forest map; (B) Fagan’s plot; (C) Sensitivity analysis; (D) Deek’s funnel plot; (E) Risk of bias summary.

# (A). Sensitivity and specificity forest map of EBNA1-IgG.

#
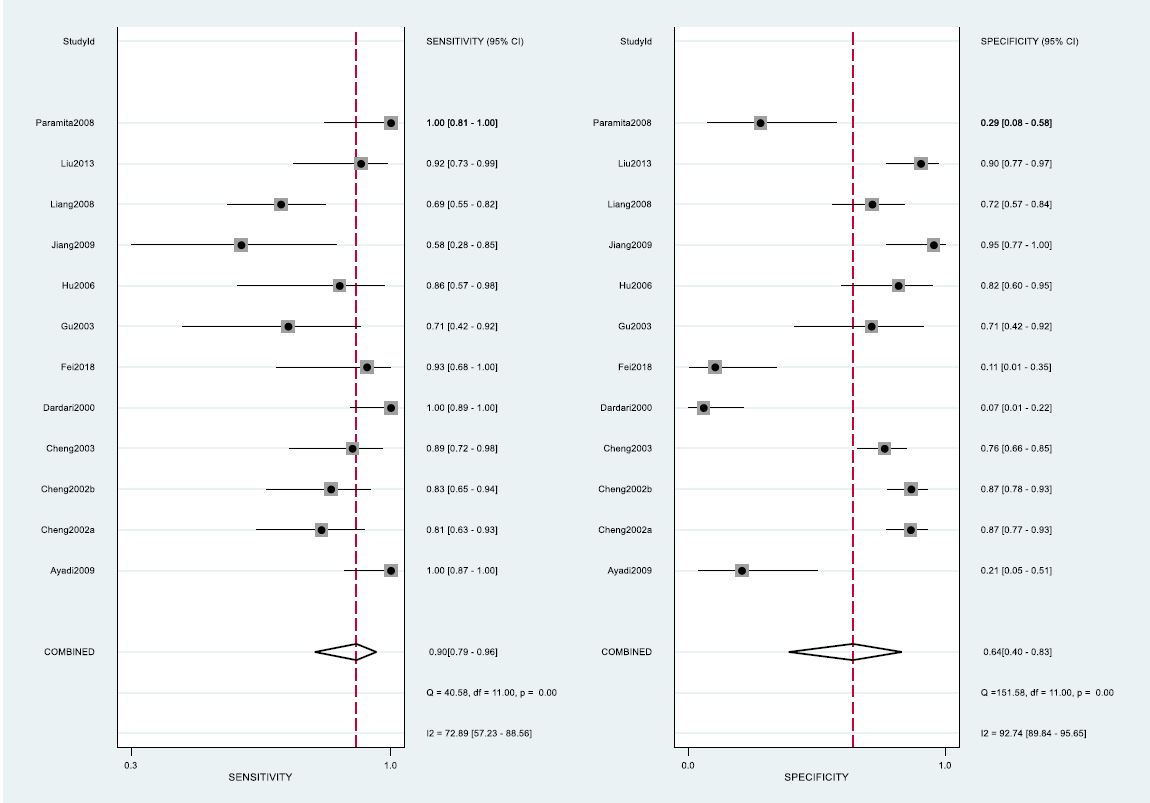


# (B). Fagan’s plot of EBNA1-IgG.

#
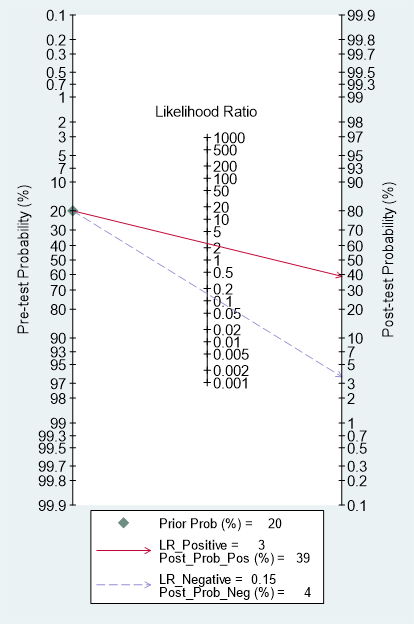


# (C). Sensitivity analysis of EBNA1-IgG.

#
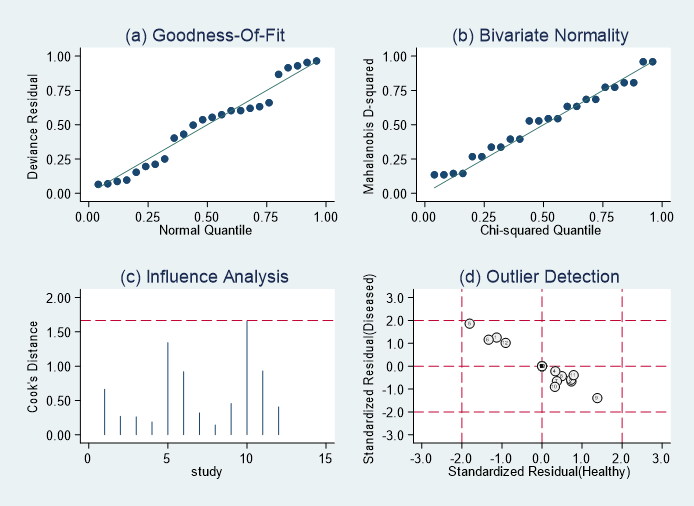


# (D). Deek’s funnel plot of EBNA1-IgA.

#
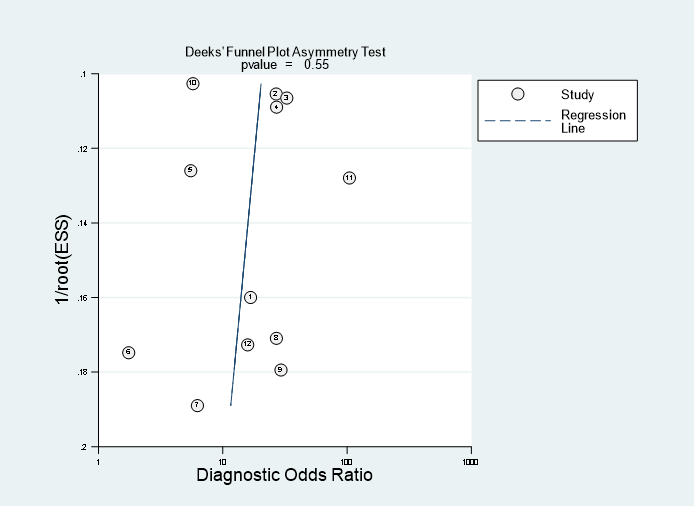


#
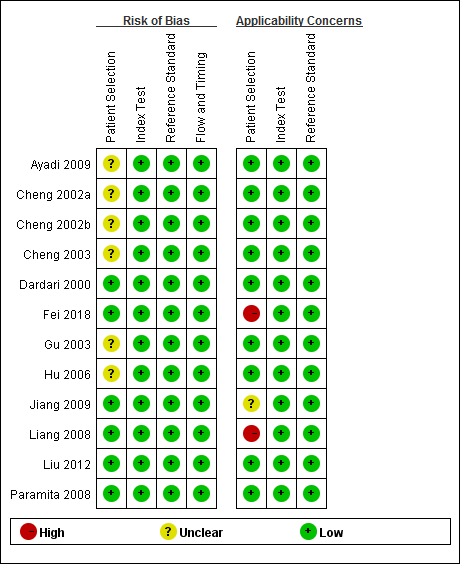


# (E). Risk of bias summary of EBNA1-IgA.

#
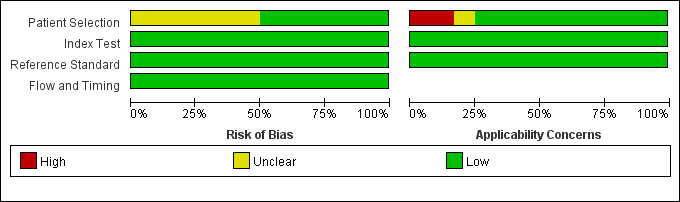


# Figure S4. Supplementary materials of EBNA1-IgG. (A) Sensitivity and specificity forest map; (B) Fagan’s plot; (C) Sensitivity analysis; (D) Deek’s funnel plot; (E) Risk of bias summary.

# (A). Sensitivity and specificity forest map of VCA-IgA.

#
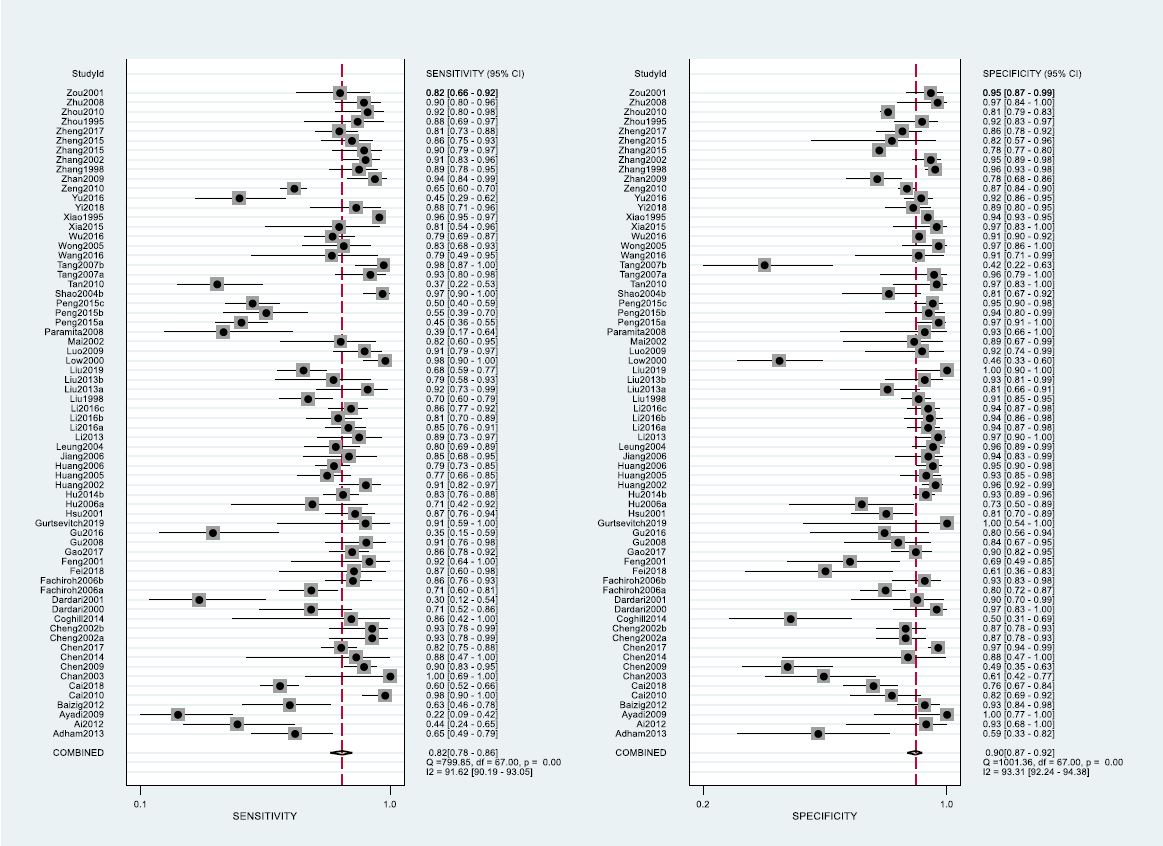


# (B). Fagan’s plot of VCA-IgA.

#
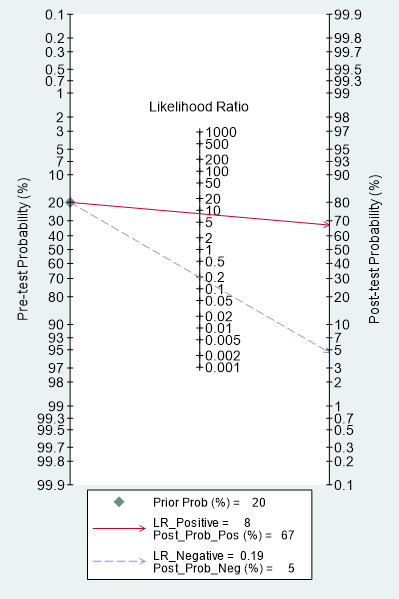


# (C). Sensitivity analysis of VCA-IgA.

#
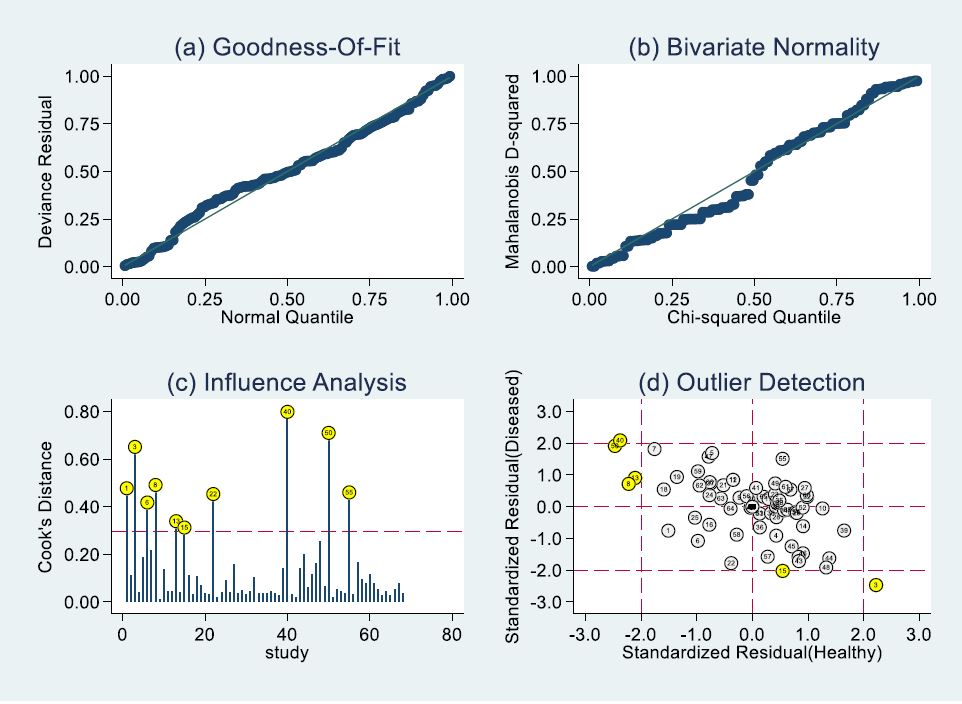


# (D). Deek’s funnel plot of VCA-IgA.

#
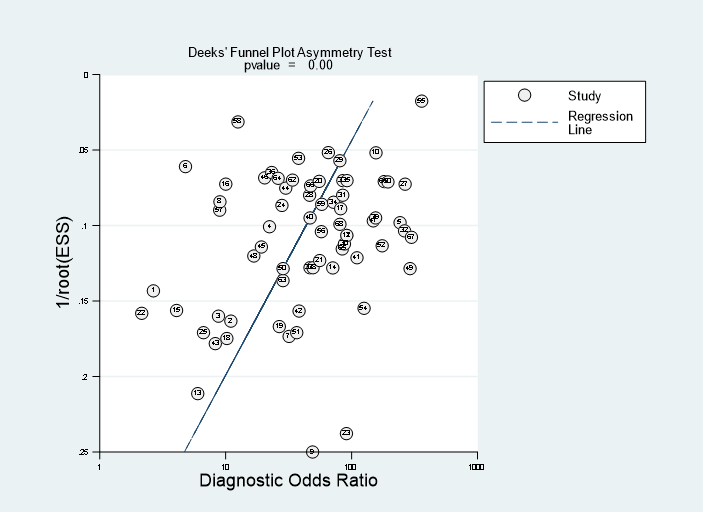


#
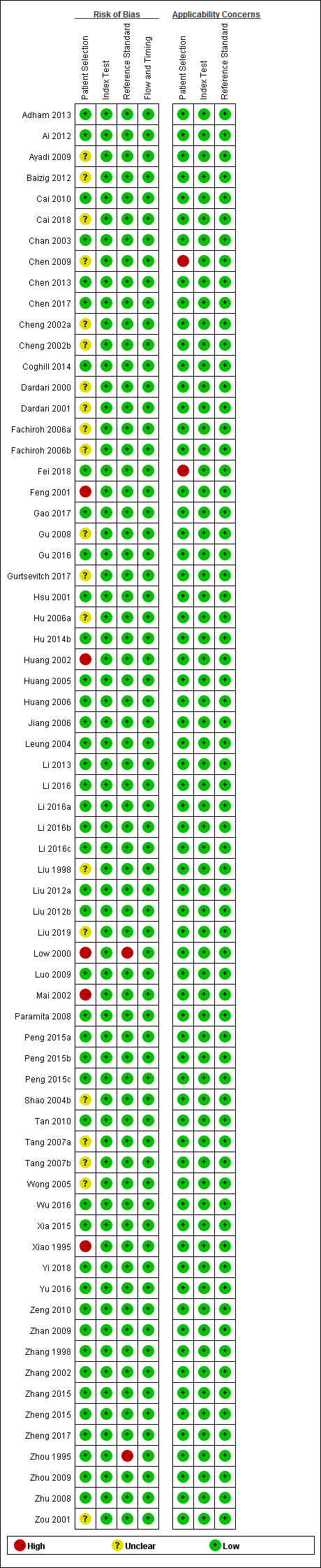


# (E). Risk of bias summary of VCA-IgA.

#
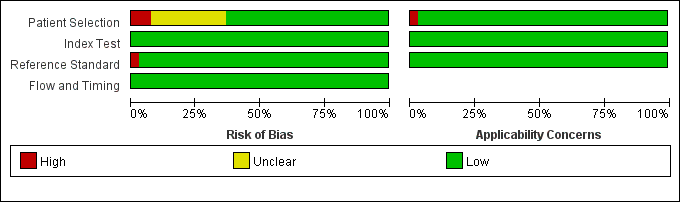


# Figure S5. Supplementary materials of VCA-IgA. (A) Sensitivity and specificity forest map; (B) Fagan’s plot; (C) Sensitivity analysis; (D) Deek’s funnel plot; (E) Risk of bias summary.

# (A). Sensitivity and specificity forest map of VCA-IgG.

#
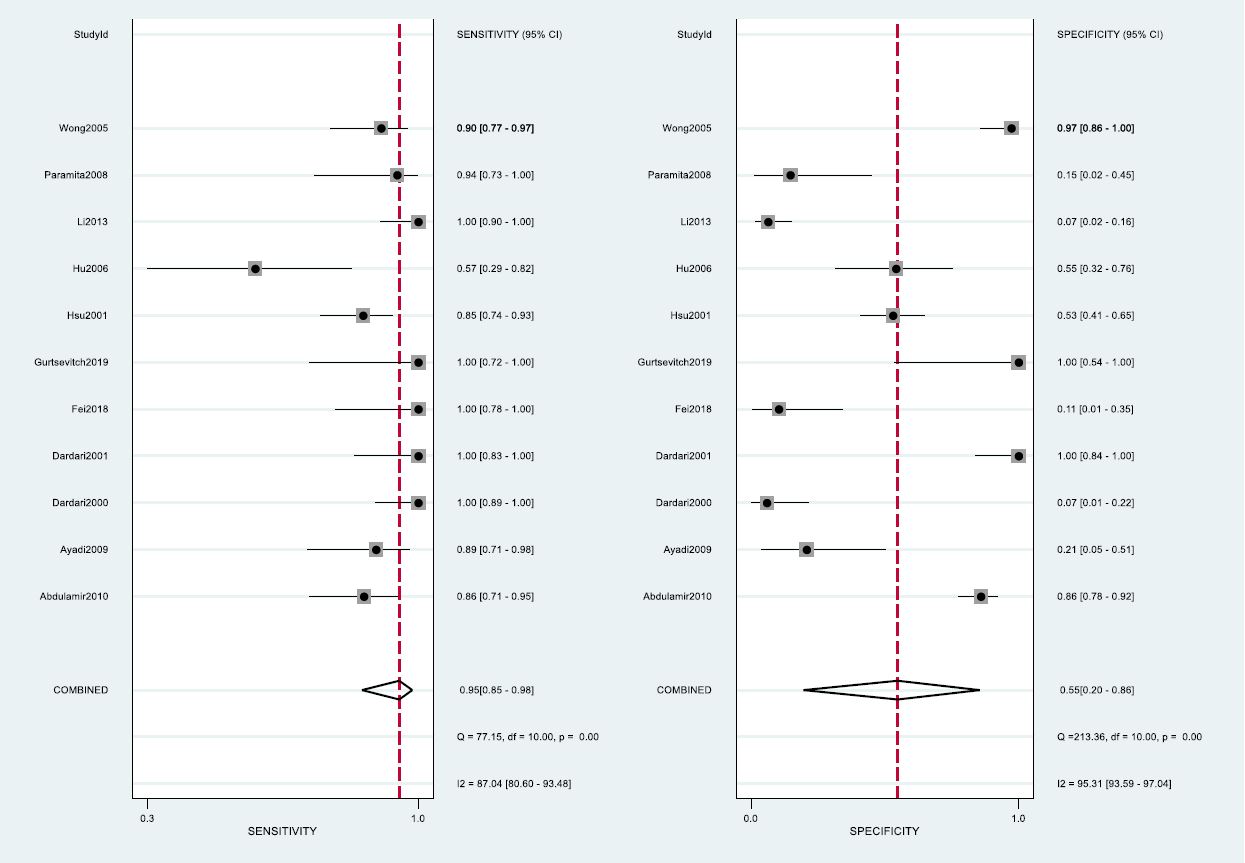


# (B). Fagan’s plot of VCA-IgG.

#
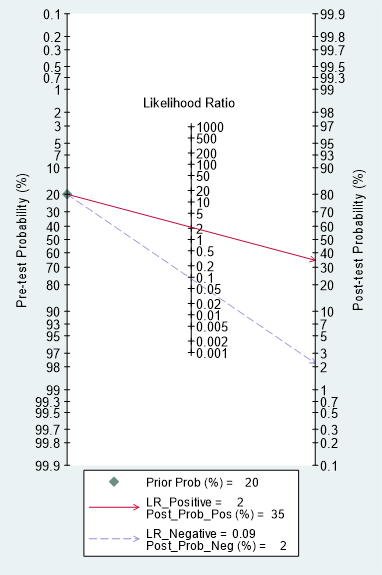


# (C). Sensitivity analysis of VCA-IgG.

#
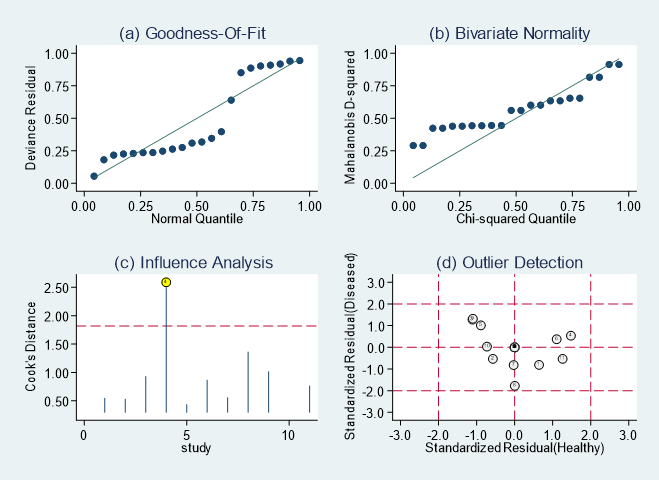


# (D). Deek’s funnel plot of VCA-IgG.

#
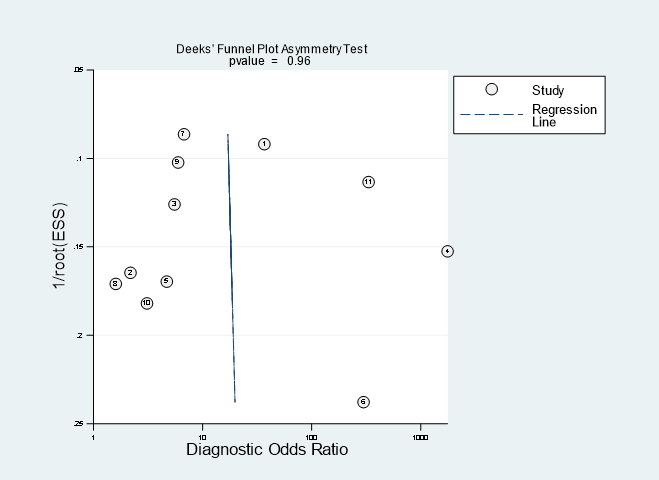


#
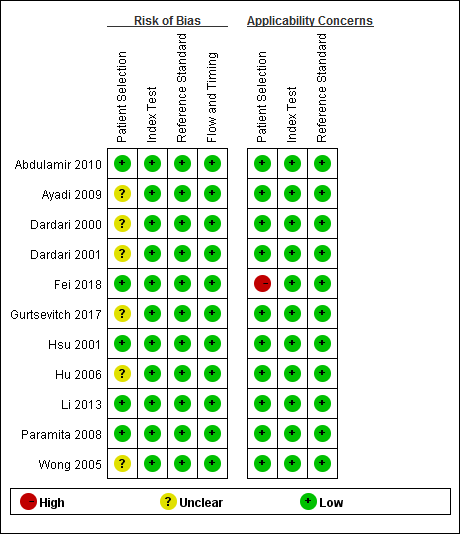


# (E). Risk of bias summary of VCA-IgG.

#
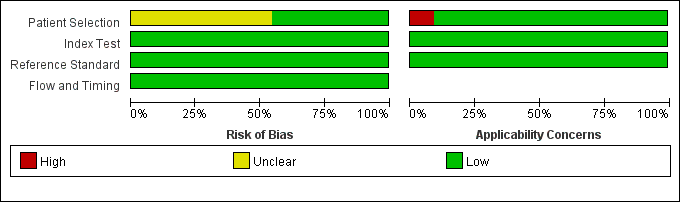


# Figure S6. Supplementary materials of VCA-IgG. (A) Sensitivity and specificity forest map; (B) Fagan’s plot; (C) Sensitivity analysis; (D) Deek’s funnel plot; (E) Risk of bias summary.

# (A). Sensitivity and specificity forest map of Rta-IgG.

#
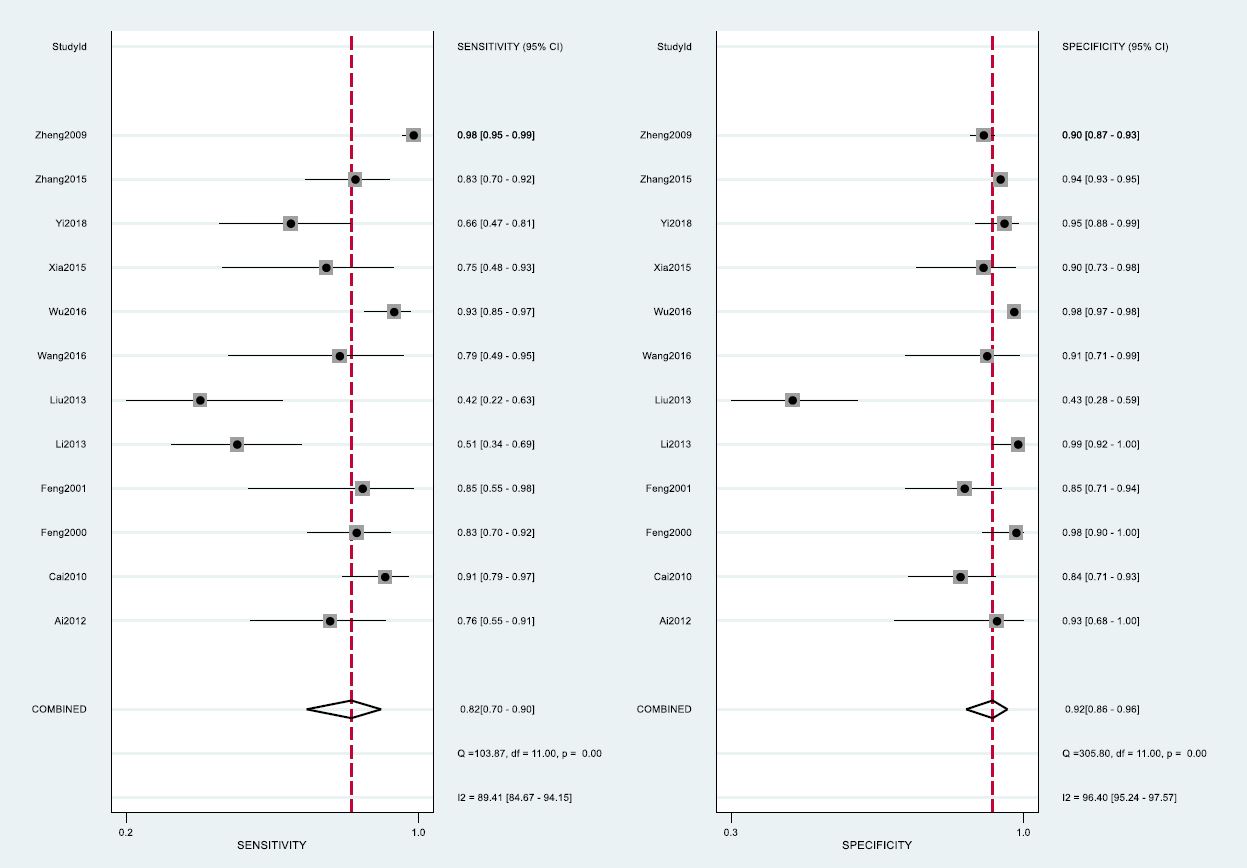


# (B). Fagan’s plot of Rta-IgG.

#
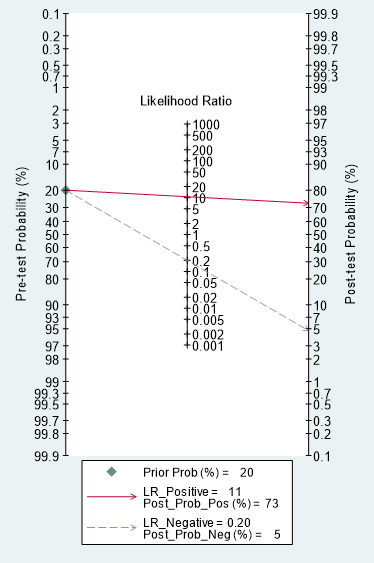


# (C). Sensitivity analysis of Rta-IgG.
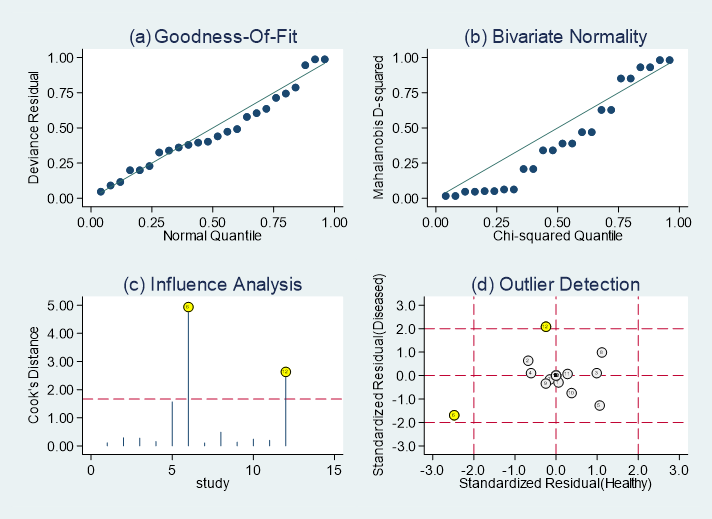


# (D). Deek’s funnel plot of Rta-IgG.

#
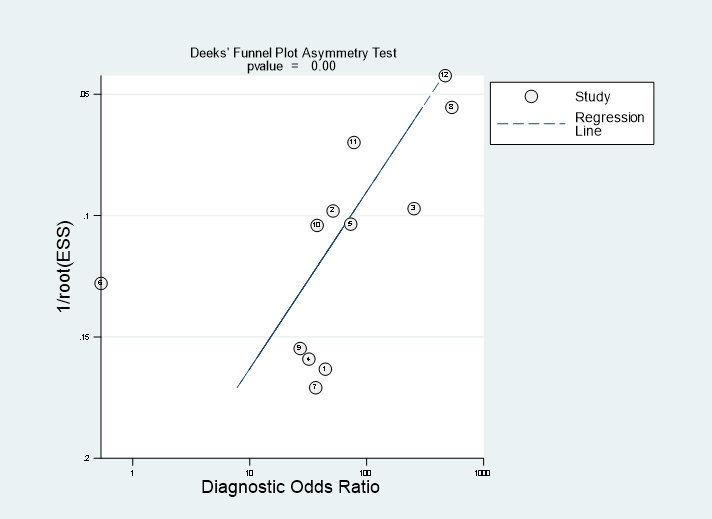


#
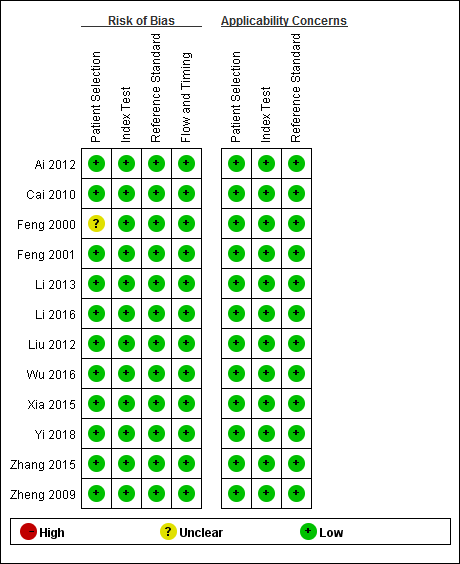
(E). Risk of bias summary of Rta-IgG.

#
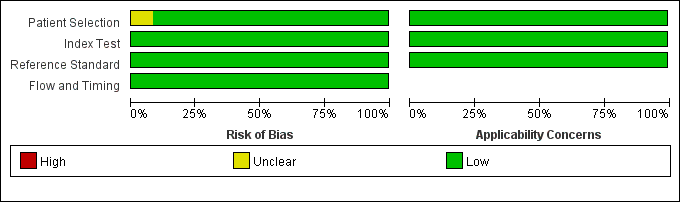


# Figure S7. Supplementary materials of Rta-IgG. (A) Sensitivity and specificity forest map; (B) Fagan’s plot; (C) Sensitivity analysis; (D) Deek’s funnel plot; (E) Risk of bias summary.

# (A). Sensitivity and specificity forest map of Zta-IgA.

#
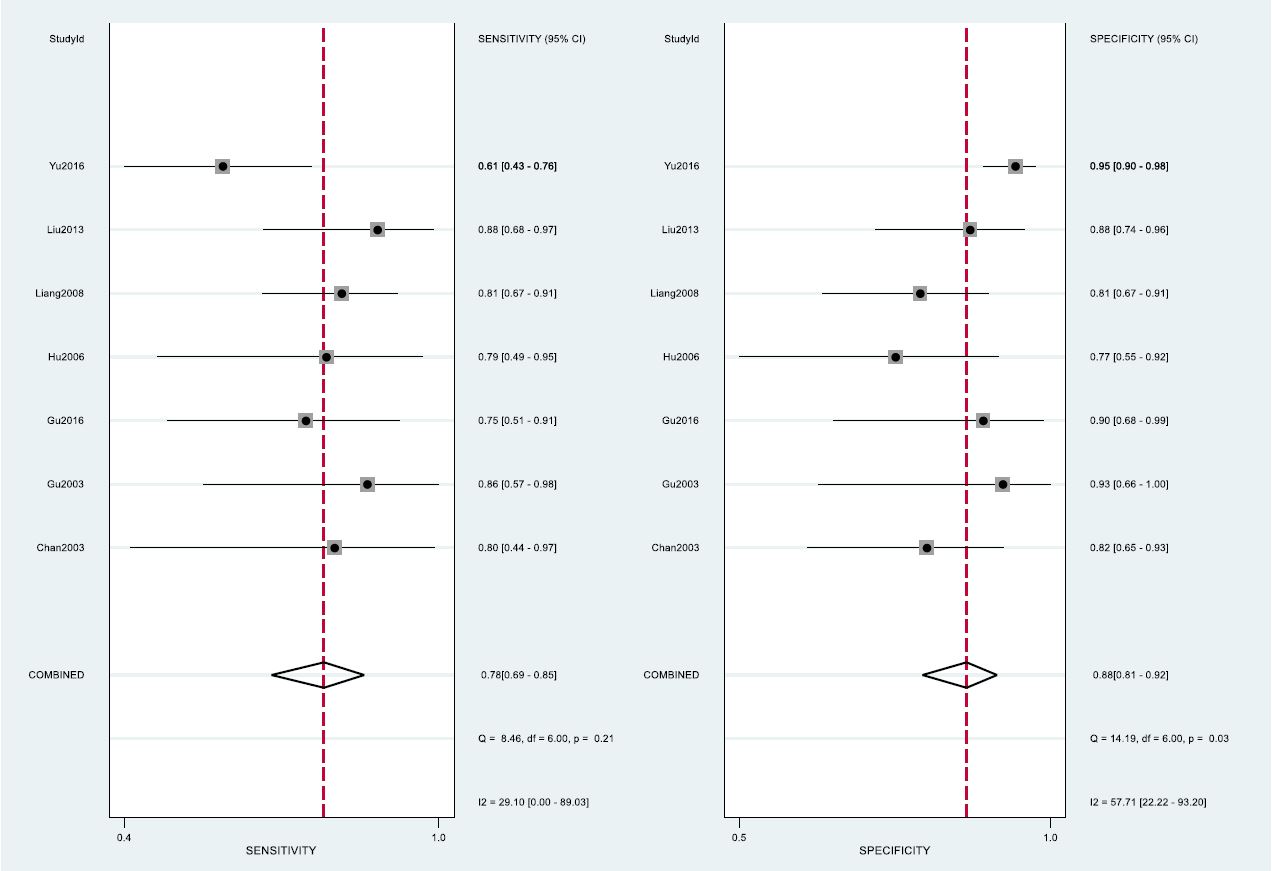


# (B). Fagan’s plot of Zta-IgA.

#
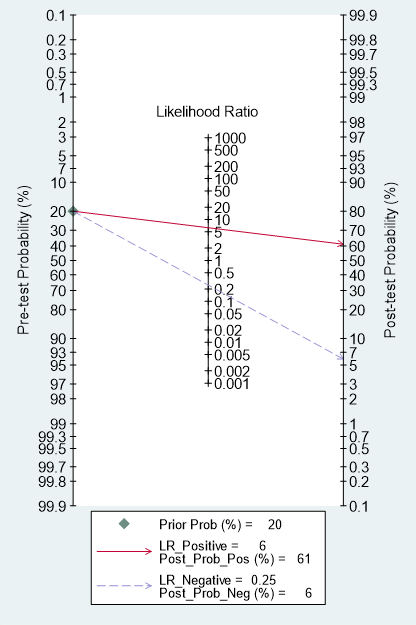


# (C). Sensitivity analysis of Zta-IgA.

#
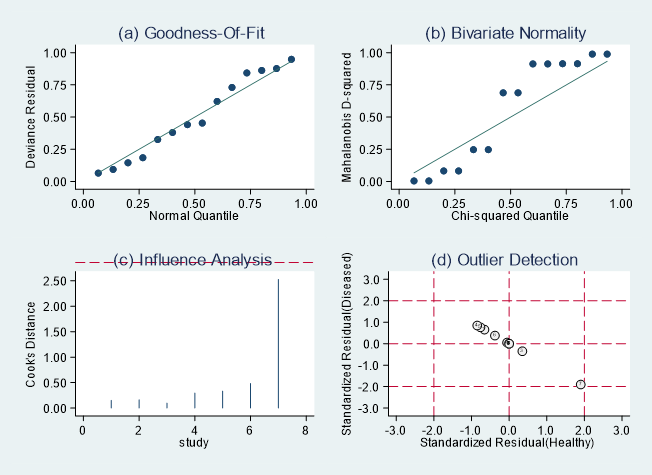


# (D). Deek’s funnel plot of Zta-IgA.

#
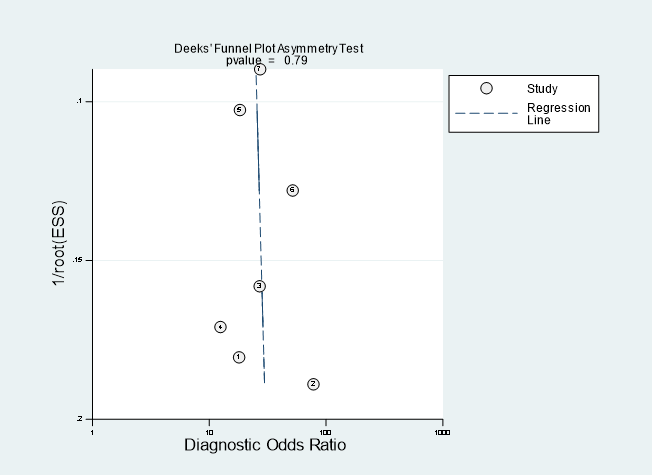


#
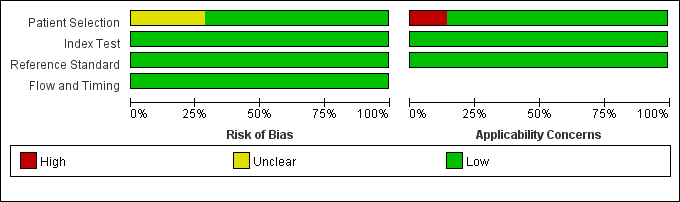

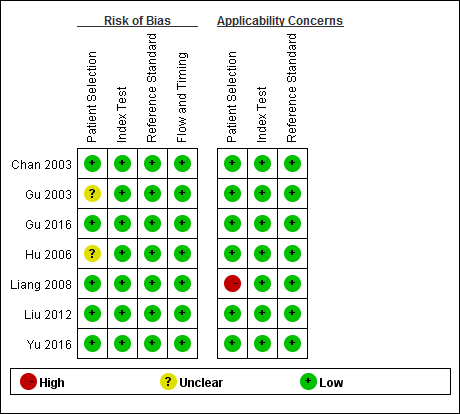
(E). Risk of bias summary of Zta-IgA.

# Figure S8. Supplementary materials of Zta-IgA. (A) Sensitivity and specificity forest map; (B) Fagan’s plot; (C) Sensitivity analysis; (D) Deek’s funnel plot; (E) Risk of bias summary.

# (A). Sensitivity and specificity forest map of Zta-IgG.

#
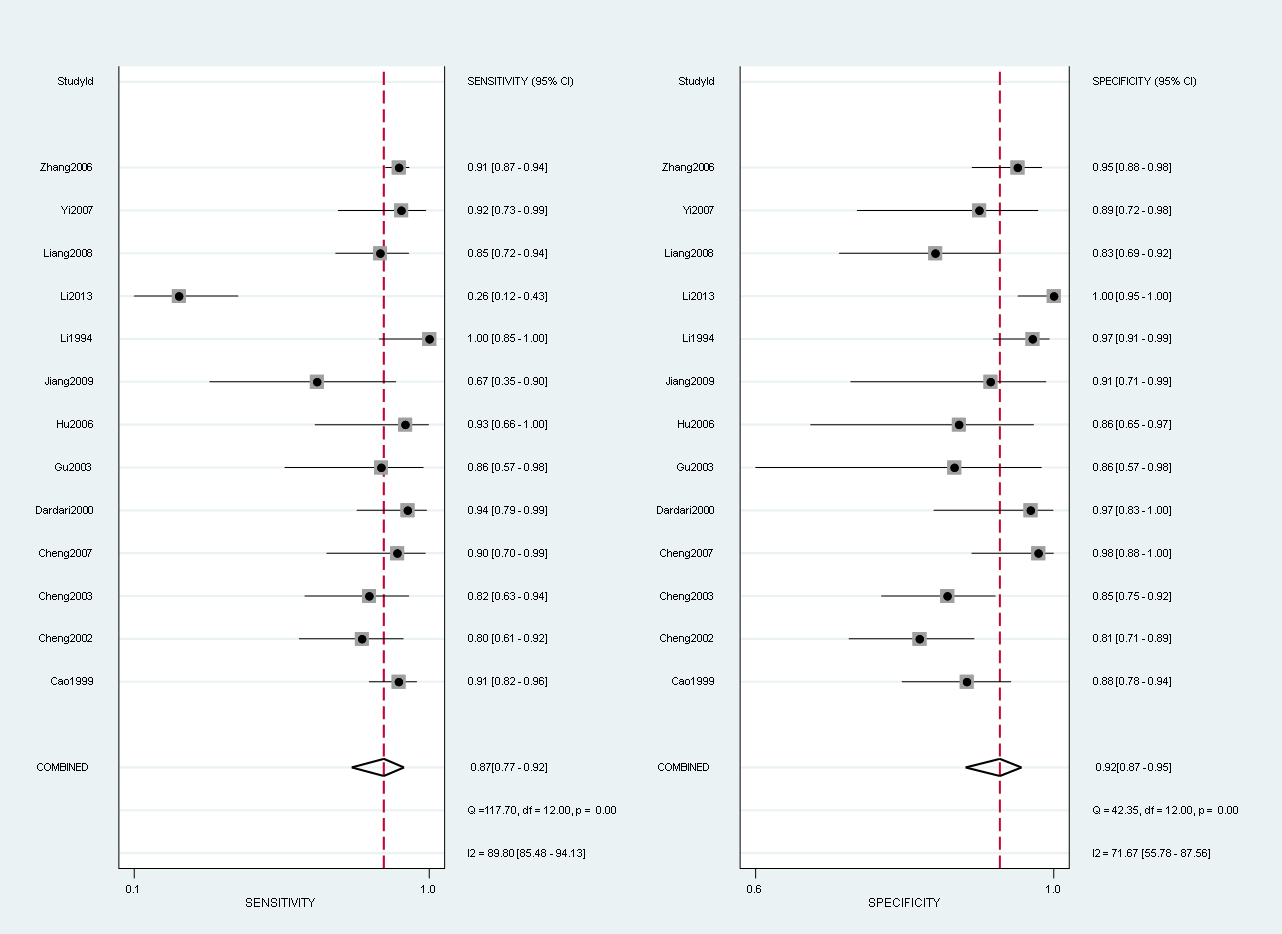


# (B). Fagan’s plot of Zta-IgG.

#
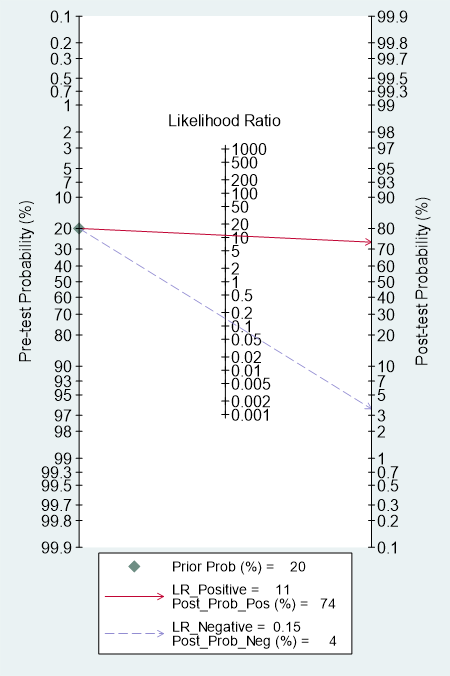


# (C). Sensitivity analysis of Zta-IgG.

#
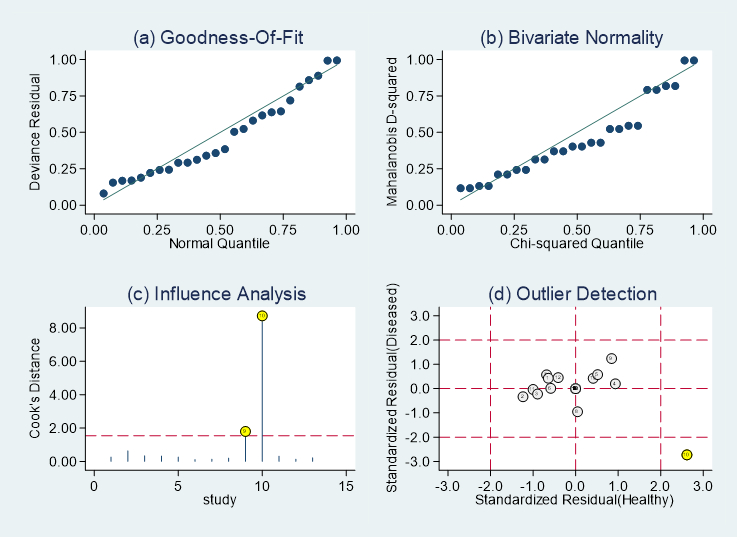


# (D). Deek’s funnel plot of Zta-IgG.

#
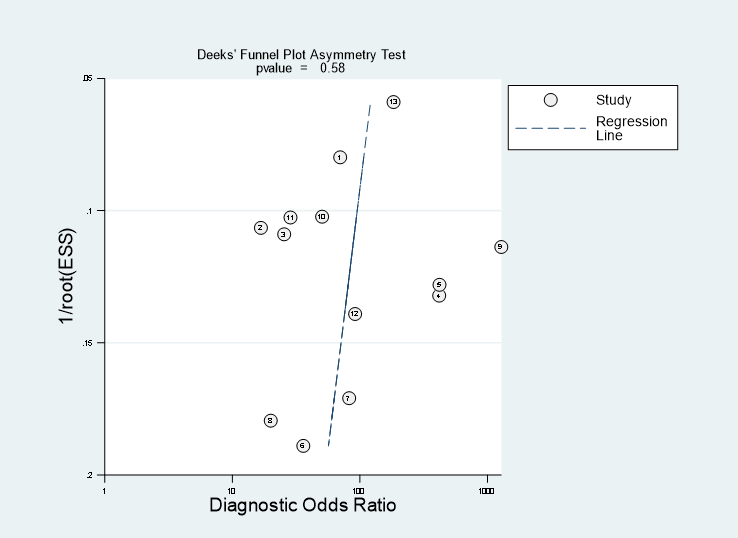


#
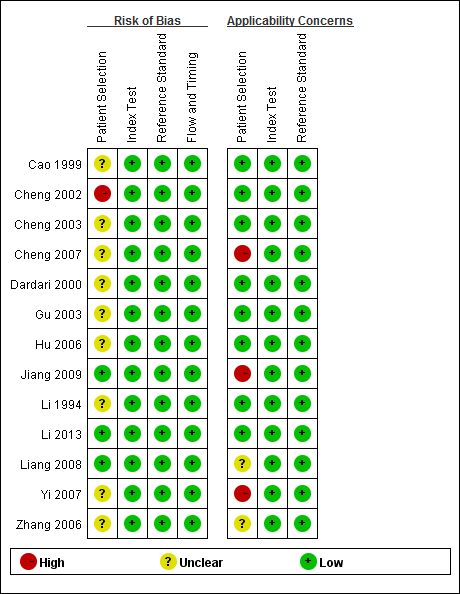
(E). Risk of bias summary of Zta-IgG.

#
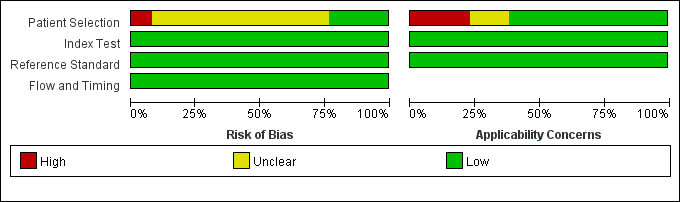


# Figure S9. Supplementary materials of Zta-IgG. (A) Sensitivity and specificity forest map; (B) Fagan’s plot; (C) Sensitivity analysis; (D) Deek’s funnel plot; (E) Risk of bias summary.

# (A). Sensitivity and specificity forest map of EBV-DNA brushings.

#
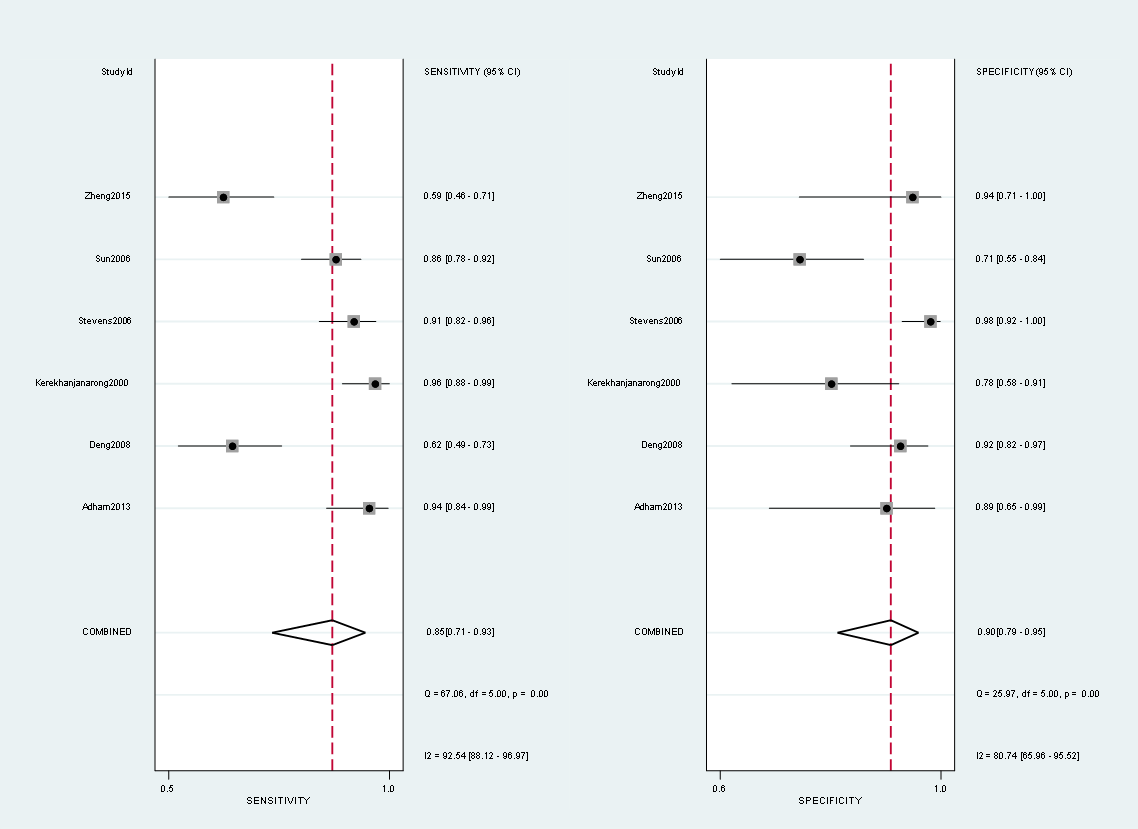


# (B). Fagan’s plot of EBV-DNA brushings.

#
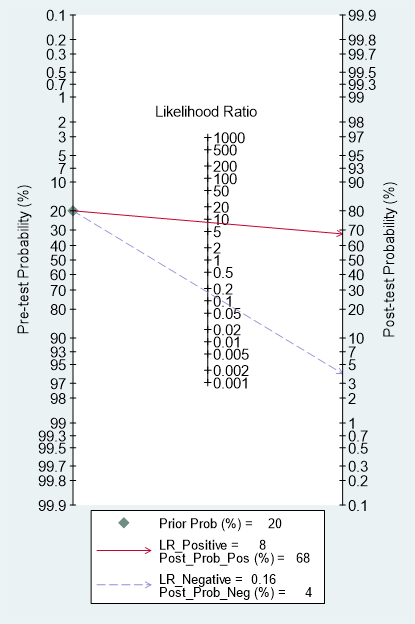


# (C). Deek’s funnel plot of EBV-DNA brushings.
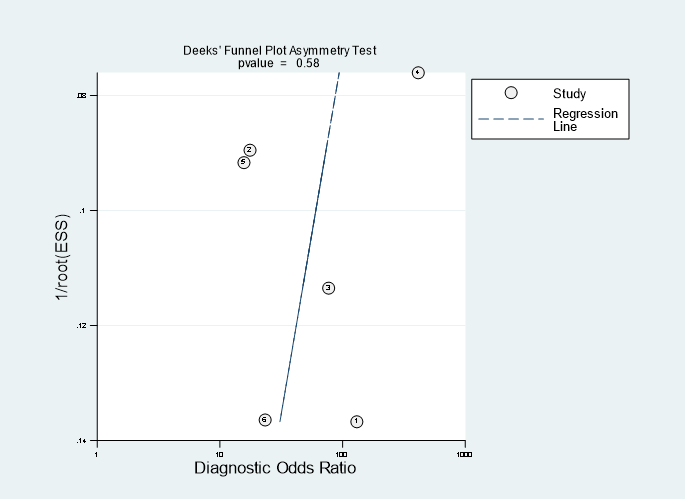


# (D). Risk of bias summary of EBV-DNA brushings.

#
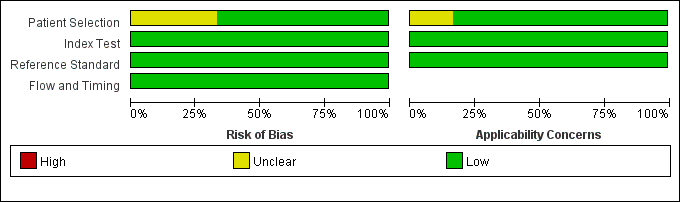

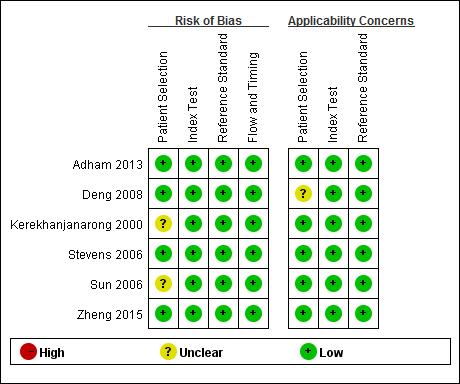


# Figure S10. Supplementary materials of EBV-DNA brushings. (A) Sensitivity and specificity forest map; (B) Fagan’s plot; (C) Deek’s funnel plot; (D) Risk of bias summary.

# (A). Sensitivity and specificity forest map of EBV-DNA plasma.


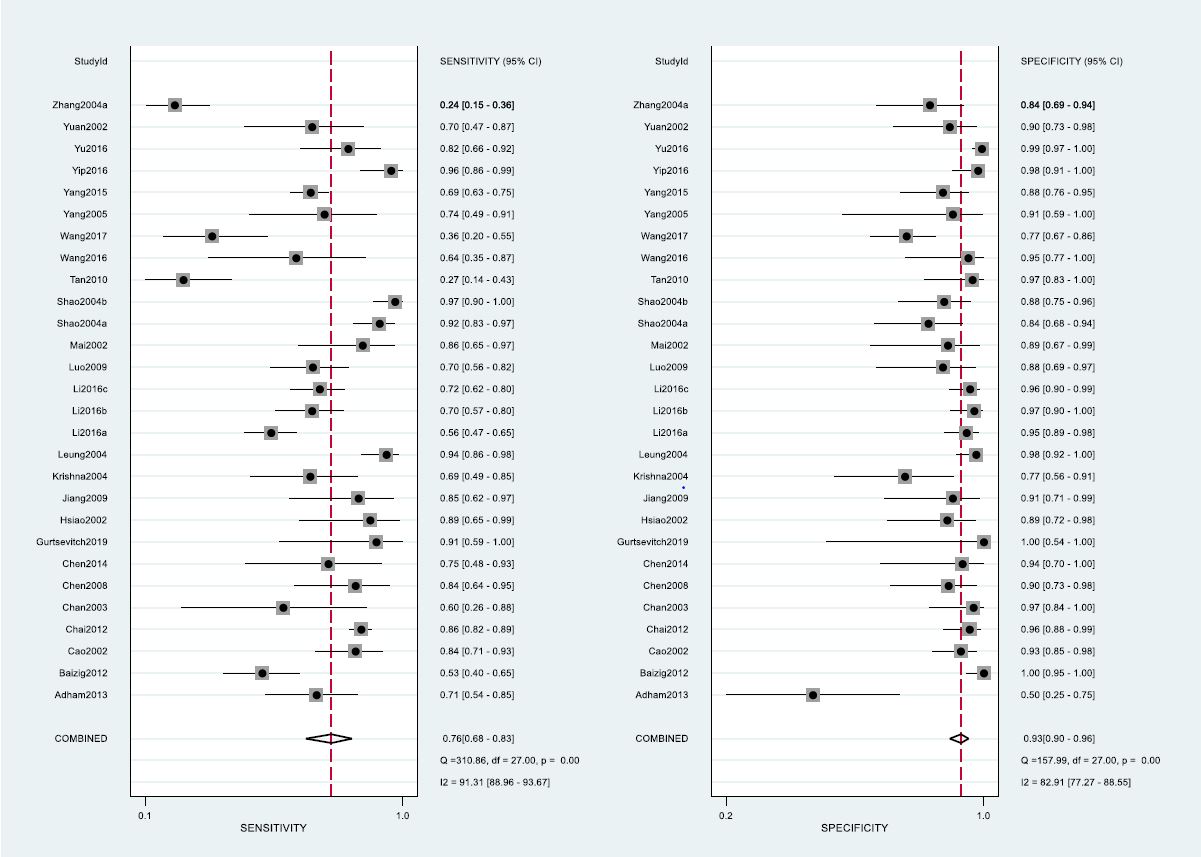


# (B). Fagan’s plot of EBV-DNA plasma.


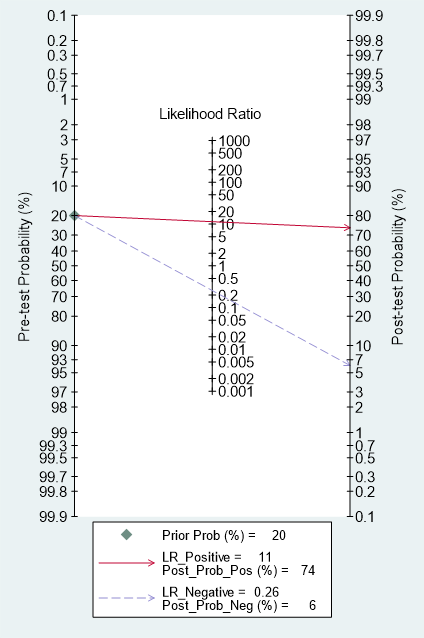


# (C). Sensitivity analysis of EBV-DNA plasma.

#
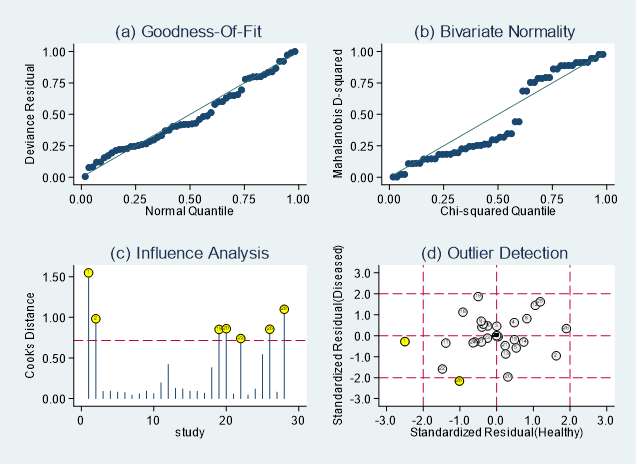


# (D). Deek’s funnel plot of EBV-DNA plasma.

#
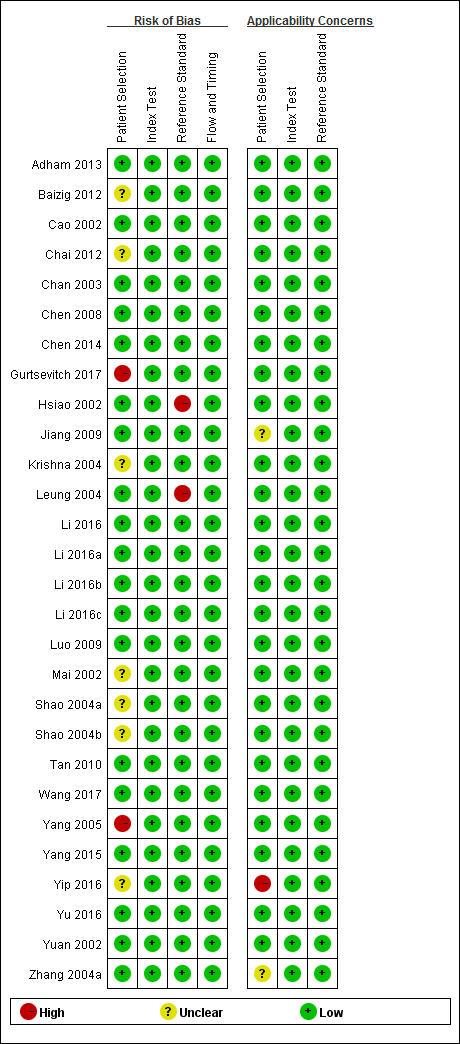

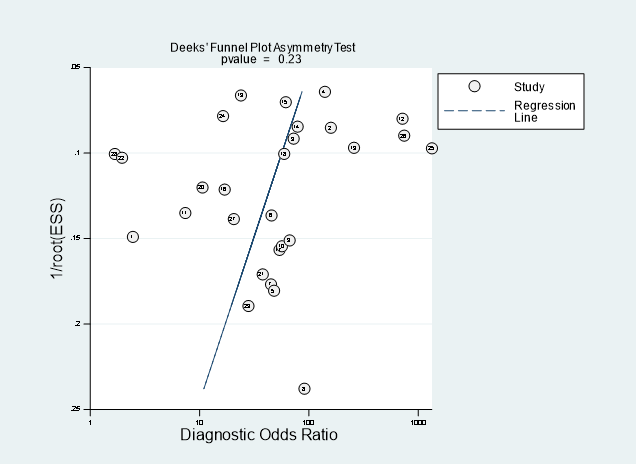


#
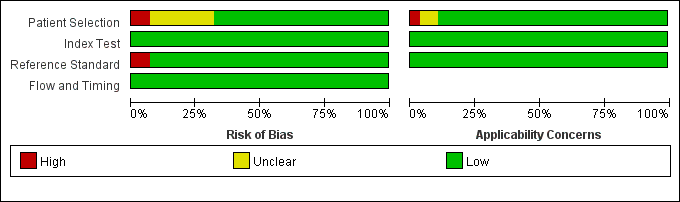
(E). Risk of bias summary of EBV-DNA plasma.

# Figure S11. Supplementary materials of EBV-DNA plasma. (A) Sensitivity and specificity forest map; (B) Fagan’s plot; (C) Sensitivity analysis; (D) Deek’s funnel plot; (E) Risk of bias summary.

# (A). Sensitivity and specificity forest map of EBV-DNA PMB.

#
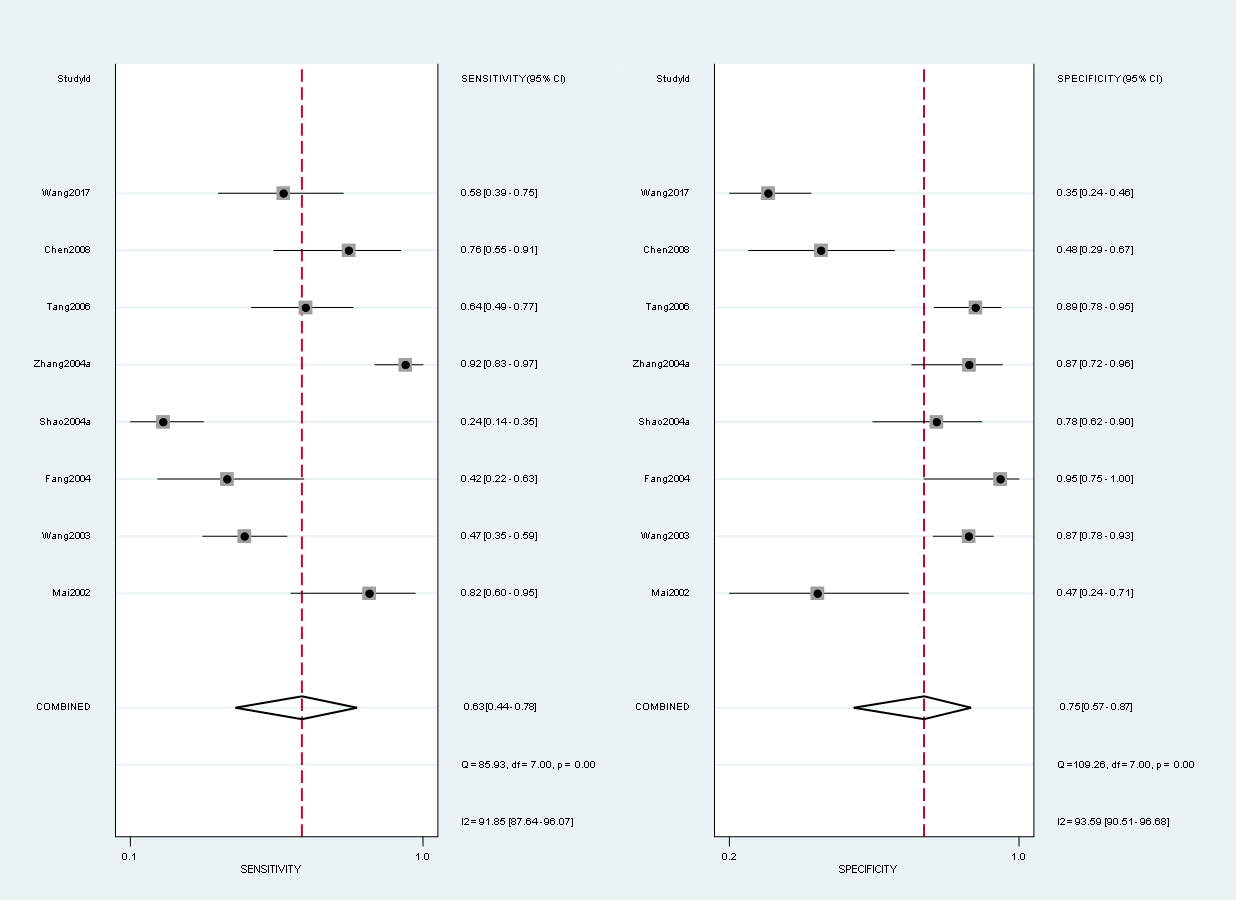


# (B). Fagan’s plot of EBV-DNA PMB.

#
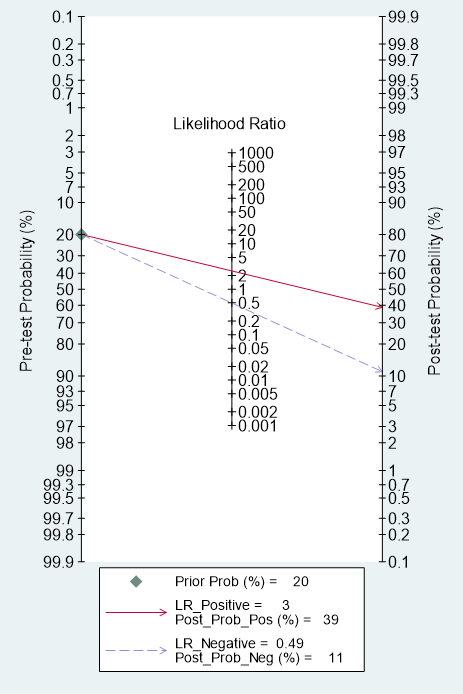


# (C). Sensitivity analysis of EBV-DNA PMB.

#
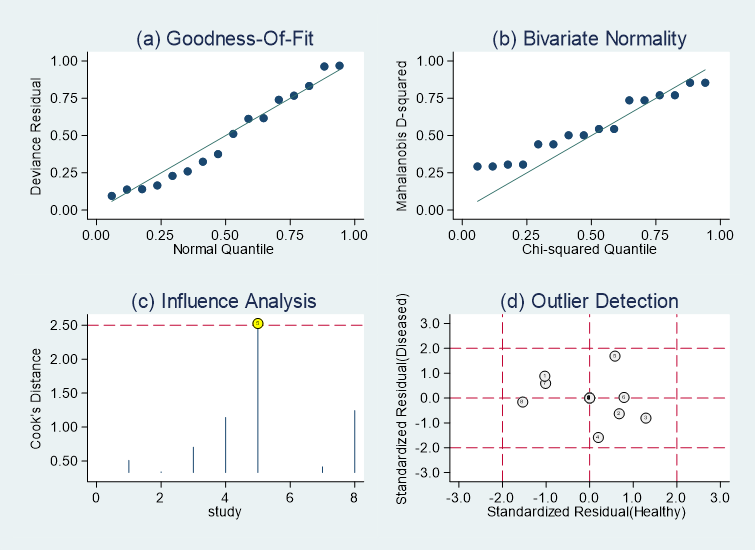


# (D). Deek’s funnel plot of EBV-DNA PMB.

#
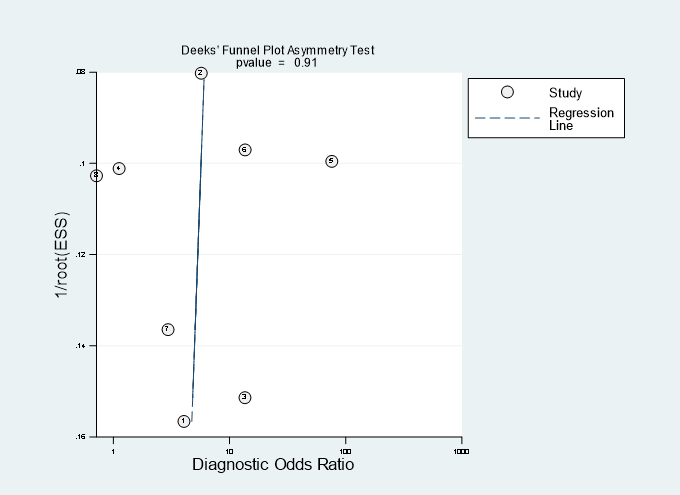


#
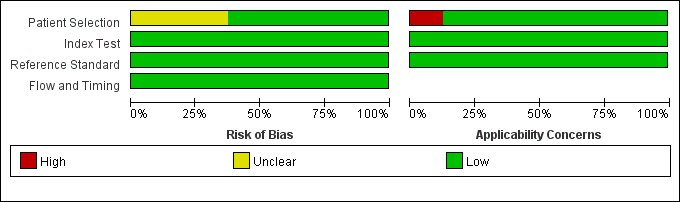

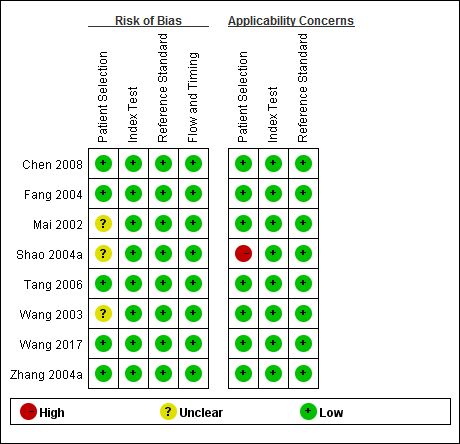
(E). Risk of bias summary of EBV-DNA PMB.

# Figure S12. Supplementary materials of EBV-DNA PMB. (A) Sensitivity and specificity forest map; (B) Fagan’s plot; (C) Sensitivity analysis; (D) Deek’s funnel plot; (E) Risk of bias summary.

# (A). Sensitivity and specificity forest map of HSP70.

#
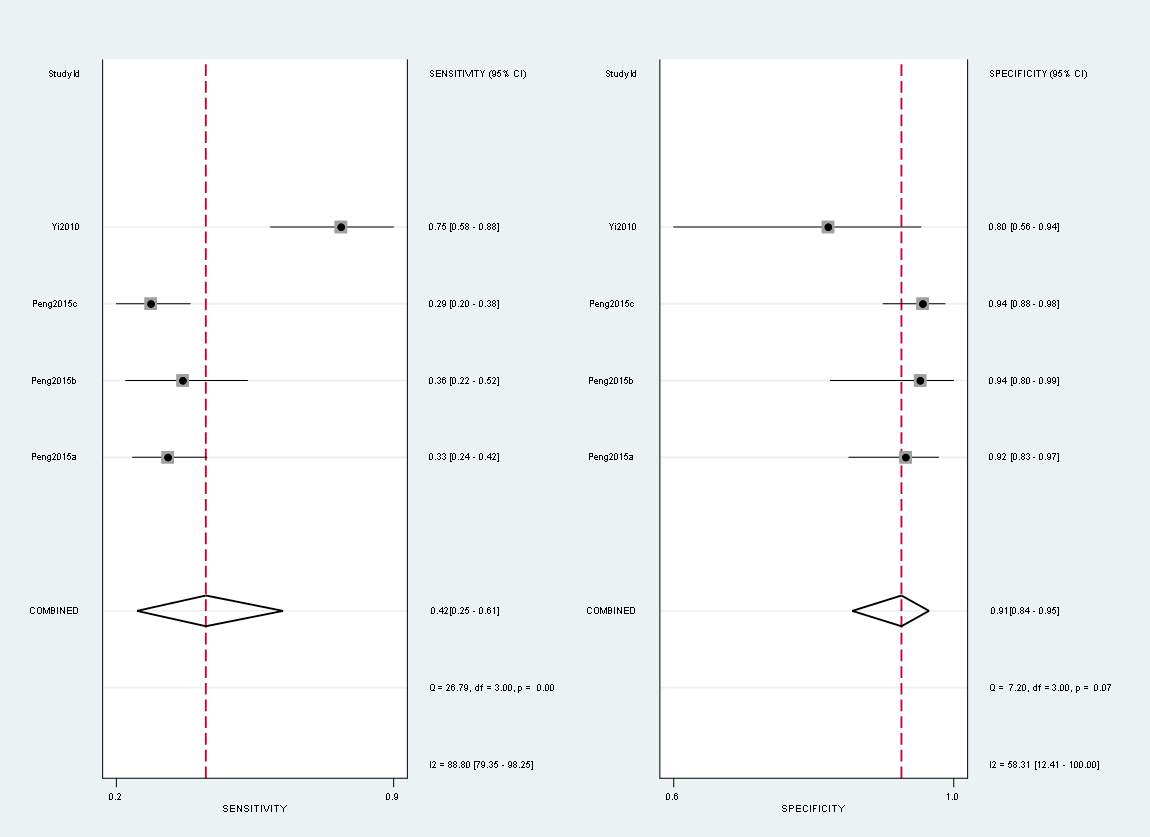


# (B). Fagan’s plot of HSP70.

#
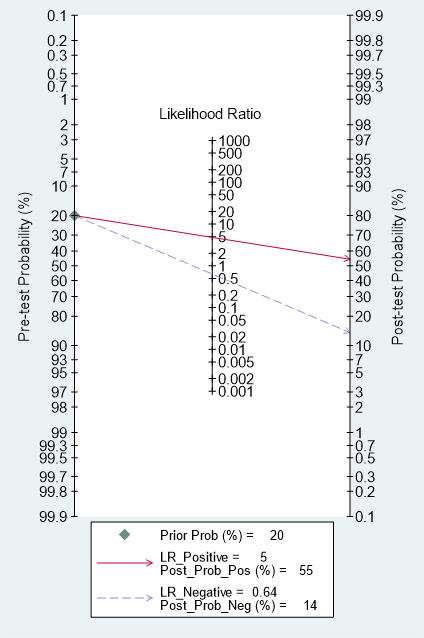


# (C). Sensitivity analysis of HSP70.

#
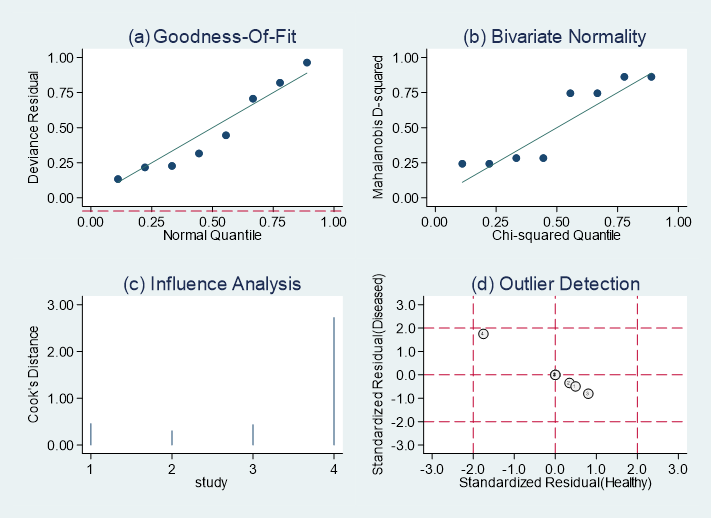


# (D). Deek’s funnel plot of HSP70.

#
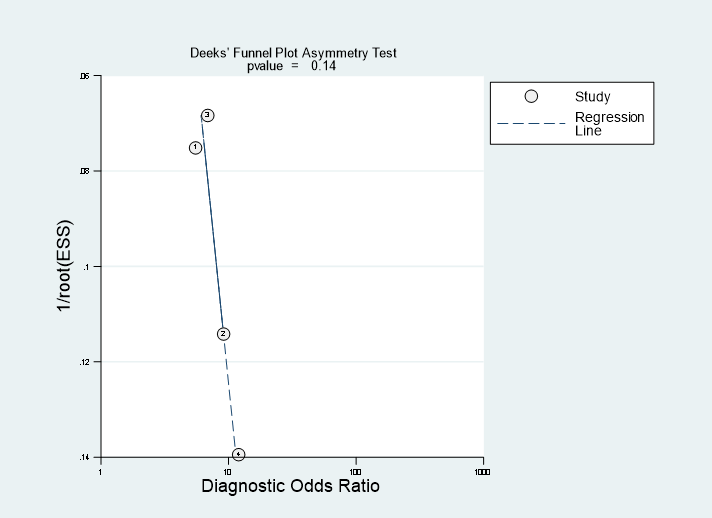


#
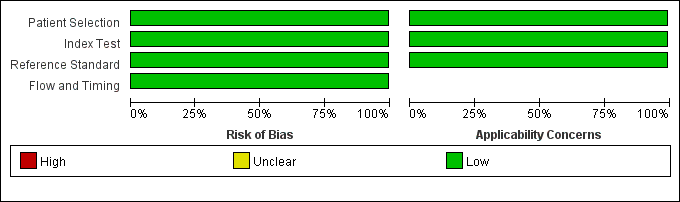

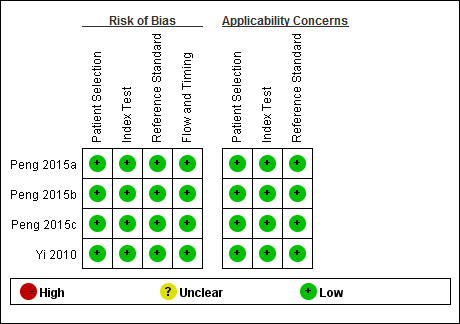
(E). Risk of bias summary of HSP70.

# Figure S13. Supplementary materials of HSP70. (A) Sensitivity and specificity forest map; (B) Fagan’s plot; (C) Sensitivity analysis; (D) Deek’s funnel plot; (E) Risk of bias summary.

# (A). Sensitivity and specificity forest map of SA.

#
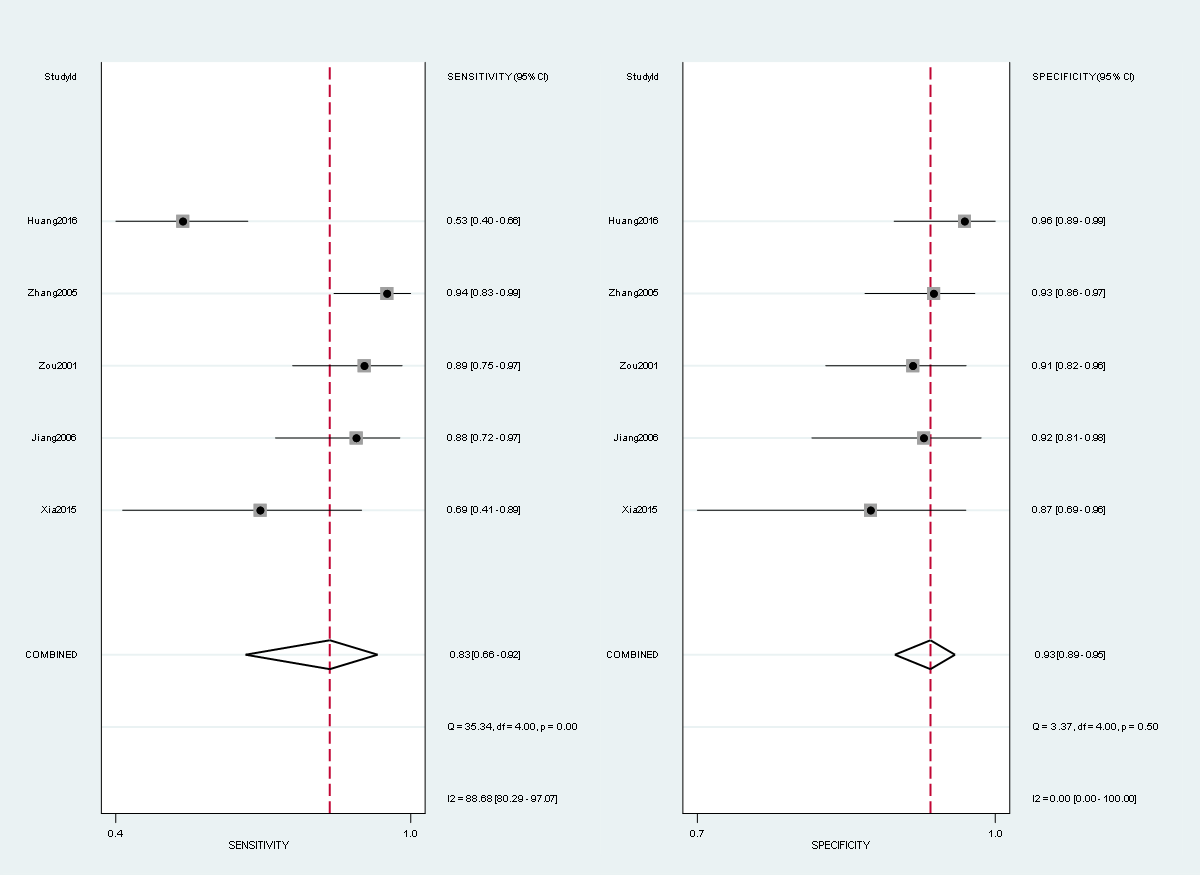


# (B). Fagan’s plot of SA.

#
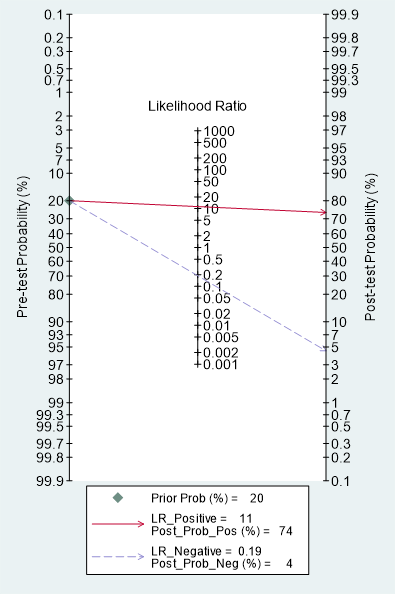


# (C). Sensitivity analysis of SA.

#
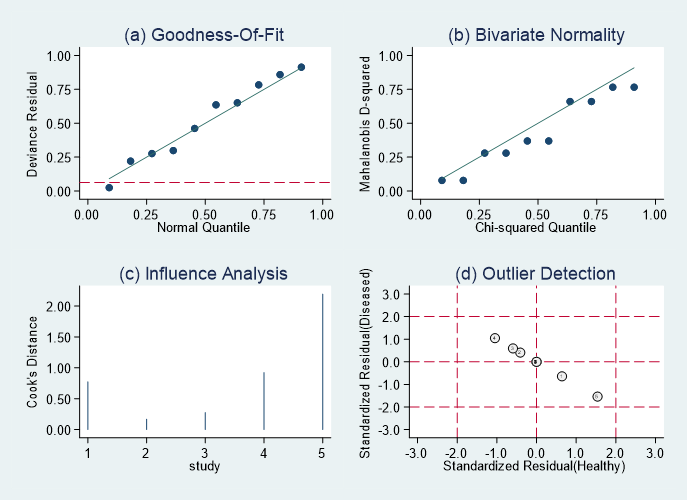


# (D). Deek’s funnel plot of SA.

#
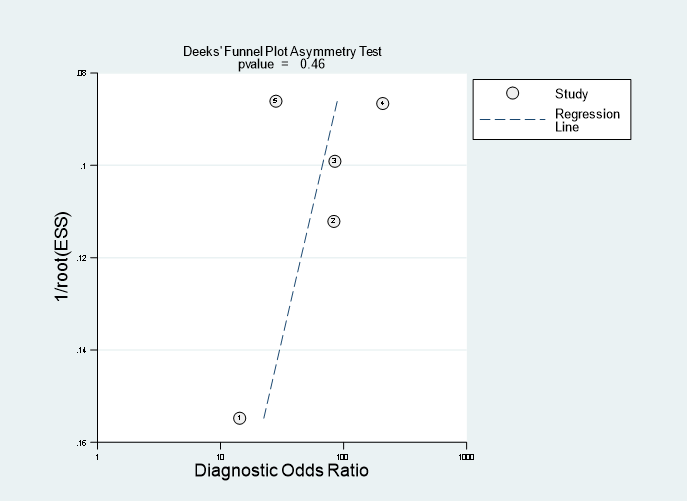


#
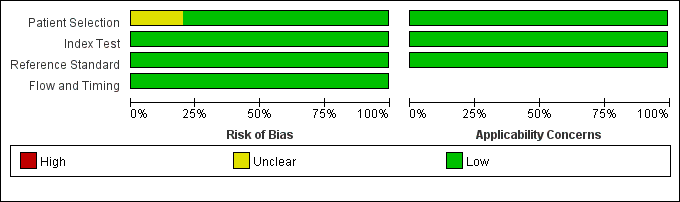

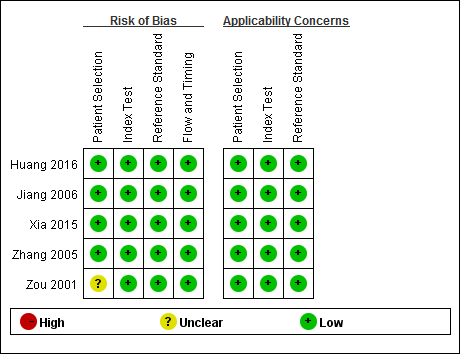
(E). Risk of bias summary of SA.

# Figure S14. Supplementary materials of SA. (A) Sensitivity and specificity forest map; (B) Fagan’s plot; (C) Sensitivity analysis; (D) Deek’s funnel plot; (E) Risk of bias summary.

**Table S1.** Main characteristics of the 101 included articles.

| Author + Year | Sample Source | Included markers | Sample Size | **Nb of Patients** | Nb of Health | Average Age of Patients | Sex Ratio of Patients (% Male) |
| --- | --- | --- | --- | --- | --- | --- | --- |
| Abdulamir 2010 | Malaysia | VCA-IgG | 142 | 42 | 100 | 54 | 78.5% |
| Adham 2013 | Indonesia | EBNA1-IgA, EBV-DNA brushings, EBV-DNA plasma, VCA-IgA | 244 | 176 | 68 | 48 | 67.2% |
| Ai 2012 | China | EA-IgG, EBNA1-IgA, Rta-IgG, VCA-IgA | 160 | 100 | 60 | 45 | 78% |
| Akbar 2016 | Indonesia | EA-IgA | 200 | 100 | 100 | - | - |
| Ayadi 2009 | Tunisia | EBNA1-IgA, EBNA1-IgG, VCA-IgA, VCA-IgG | 164 | 108 | 56 | - | - |
| Baizig 2012 | Tunisia | EBV-DNA plasma, VCA-IgA | 244 | 104 | 140 | - | - |
| Cai 2010 | China | EA-IgA, EBNA1-IgA, Rta-IgG, VCA-IgA | 416 | 212 | 204 | 48 | 73.6% |
| Cai 2018 | China | VCA-IgA | 300 | 198 | 102 | - | - |
| Cao 1999 | China | Zta-IgG | 157 | 77 | 80 | - | - |
| Cao 2002 | China | EBV-DNA plasma | 124 | 50 | 74 | - | - |
| Chai 2012 | Malaysia | EBV-DNA plasma | 462 | 390 | 72 | 50.5 | - |
| Chan 2003 | China | EA-IgA, EBNA1-IgA, EBV-DNA plasma, VCA-IgA, Zta-IgA | 215 | 50 | 165 | - | - |
| Chen 2008 | China | EBV-DNA plasma, EBV-DNA PMB, | 108 | 50 | 58 | 46.8 | - |
| Chen 2009 | China | EA-IgA, VCA-IgA, | 330 | 228 | 102 | 48.8 | 75% |
| Chen 2013 | China | EA-IgA, EBV-DNA plasma, VCA-IgA | 64 | 32 | 32 | - | - |
| Chen 2017 | China | EA-IgA, VCA-IgA | 794 | 300 | 494 | - | - |
| Cheng 2002 | China | EBNA1-IgA, EBNA1-IgG, VCA-IgA, Zta-IgG | 452 | 122 | 330 | - | - |
| Cheng 2002 | China | EBNA1-IgA, EBNA1-IgG, VCA-IgA | 339 | 90 | 249 | - | 78.8% |
| Cheng 2003 | China | EBNA1-IgA, EBNA1-IgG, Zta-IgG | 339 | 84 | 255 | - | 75.3% |
| Cheng 2007 | China | EBNA1-IgA, Zta-IgG | 132 | 42 | 90 | - | 85.4% |
| Coghill 2014 | China | EA-IgA, EBNA1-IgA, VCA-IgA, | 105 | 21 | 84 | - | - |
| Coghill 2016 | China | EBNA1-IgA | 80 | 30 | 50 | 45.8 | 73.3% |
| Dardari 2000 | Morocco | EA-IgA, EA-IgG, EBNA1-IgG, VCA-IgA, VCA-IgG, Zta-IgG | 366 | 186 | 180 | - | - |
| Dardari 2001 | Morocco | EA-IgA, EA-IgG, EBNA1-IgA, VCA-IgA, VCA-IgG | 205 | 100 | 105 | - | - |
| Deng 2008 | China | EBV-DNA brushings | 125 | 65 | 60 | 49 | 66.2% |
| Fachiroh 2006 | China | EBNA1-IgA, VCA-IgA | 258 | 146 | 112 | - | - |
|  | Indonesia |  | 406 | 152 | 254 | - | - |
| Fang 2004 | China | EBV-DNA PMB | 44 | 24 | 20 | 52.7 | 75% |
| Fei 2018 | China | EA-IgG, EBNA1-IgG, VCA-IgA, VCA-IgG | 132 | 60 | 72 | 43.5 | - |
| Feng 2000 | Singapore | Rta-IgG | 106 | 53 | 53 | - | - |
| Feng 2001 | Singapore | EA-IgA, Rta-IgG, VCA-IgA | 138 | 39 | 99 | - | - |
| Gao 2017 | China | EBNA1-IgA, VCA-IgA | 400 | 200 | 200 | 43.7 | 77% |
| Gu 2003 | China | EBNA1-IgA, EBNA1-IgG, Zta-IgA, Zta-IgG | 112 | 56 | 56 | - | - |
| Gu 2008 | China | EA-IgA, EA-IgG, EBNA1-IgA, VCA-IgA | 264 | 136 | 128 | 45.8 | 71.3% |
| Gu 2016 | China | EBNA1-IgA, VCA-IgA, Zta-IgA | 120 | 60 | 60 | 51.1 | 73.3% |
| Gurtsevitch 2017 | Russia | EBV-DNA plasma, VCA-IgA, VCA-IgG | 51 | 33 | 18 | 45.6 | 61.5% |
| Hsiao 2002 | China | EBV-DNA plasma | 46 | 18 | 28 | - | - |
| Hsu 2001 | China | EA-IgA, EA-IgG, VCA-IgA, VCA-IgG | 337 | 160 | 177 | - | - |
| Hu 2006 | China | EBNA1-IgA, EBNA1-IgG, VCA-IgA, VCA-IgG, Zta-IgA, Zta-IgG | 216 | 84 | 132 | - | - |
| Hu 2014 | China | EBNA1-IgA, VCA-IgA | 800 | 300 | 500 | 45 | 73.7% |
| Huang 2002 | China | EA-IgG, VCA-IgA | 460 | 134 | 326 | - | 77.6% |
| Huang 2005 | China | VCA-IgA | 157 | 82 | 75 | 51 | 75.6% |
| Huang 2006 | China | VCA-IgA | 316 | 184 | 132 | - | 70.7% |
| Huang 2016 | China | SA | 137 | 60 | 77 | 51.1 | 73.3% |
| Jiang 2006 | China | SA, VCA-IgA | 166 | 66 | 100 | 47 | 75.8% |
| Jiang 2009 | China | EBNA1-IgA, EBNA1-IgG, EBV-DNA plasma, Zta-IgG | 144 | 56 | 88 | 46 | 72.8% |
| Kerekhanjanarong 2000 | Thailand | EBV-DNA brushings | 96 | 69 | 27 | - | - |
| Krishna 2004 | India | EBV-DNA plasma | 55 | 29 | 26 | - | 62% |
| Leung 2004 | China | EBV-DNA plasma, VCA-IgA | 318 | 140 | 178 | - | - |
| Li 1994 | China | Zta-IgG | 121 | 23 | 98 | - | - |
| Li 2013 | China | Rta-IgG, VCA-IgA, VCA-IgG, Zta-IgG | 420 | 140 | 280 | 45.5 | 68.6 |
| Li 2016a | China | EA-IgG, EBV-DNA plasma, Rta-IgG, VCA-IgA | 144 | 56 | 88 | 43.6 | 48.2 |
| Li 2016b | China | EBV-DNA plasma, VCA-IgA | 686 | 435 | 341 | - | - |
| Li 2016c | China | EBV-DNA plasma, VCA-IgA | 406 | 208 | 198 | - | - |
| Liang 2008 | China | EBNA1-IgA, EBNA1-IgG, Zta-IgA, Zta-IgG | 380 | 193 | 187 | 46 | 74.1% |
| Liu 1998 | China | VCA-IgA | 258 | 93 | 165 | - | - |
| Liu 2012 | China | EA-IgA, EBNA1-IgA, Rta-IgG, VCA-IgA, Zta-IgA | 528 | 192 | 336 | - | - |
| Liu 2019 | China | VCA-IgA | 146 | 110 | 36 | 45 | 68.5% |
| Low 2000 | Singapore | EA-IgA, VCA-IgA | 222 | 110 | 112 | 47.6 | 70.9% |
| Luo 2009 | China | EA-IgA, EBV-DNA plasma, VCA-IgA | 234 | 159 | 75 | 49 | 72.9% |
| Mai 2002 | China | EBV-DNA plasma, EBV-DNA PMB, VCA-IgA | 123 | 66 | 57 | - | - |
| Paramita 2008 | Indonesia | EA-IgA, EA-IgG, EBNA1-IgA, EBNA1-IgG, VCA-IgA, VCA-IgG | 190 | 108 | 82 | - | - |
| Peng 2015a | China | HSP70, VCA-IgA | 428 | 210 | 218 | 51 | 71.4% |
| Peng 2015b | China | HSP70, VCA-IgA | 524 | 308 | 216 | - | - |
| Shao2004a | China | EBV-DNA plasma, EBV-DNA PMB | 221 | 147 | 74 | - | 27.9% |
| Shao2004b | China | EBV-DNA plasma, VCA-IgA | 226 | 140 | 86 | - | - |
| Stevens 2006 | Indonesia | EBV-DNA brushings | 173 | 85 | 88 | - | - |
| Sun 2006 | China | EBV-DNA brushings | 144 | 102 | 42 | - | 64.6% |
| Tan 2010 | China | EA-IgA, EBV-DNA plasma, VCA-IgA | 213 | 123 | 90 | 48 | 74.8% |
| Tang 2006 | China | EBV-DNA PMB | 108 | 47 | 61 | 45 | 65.9% |
| Tang 2007 | China | EA-IgA, VCA-IgA | 260 | 164 | 96 | - | - |
| Wang 2003 | China | EBV-DNA PMB | 156 | 73 | 83 | 46 | 72.6% |
| Wang2017 | China | EBV-DNA plasma, EBV-DNA PMB | 234 | 66 | 168 | - | - |
| Wong 2005 | Malaysia | EA-IgA, EA-IgG, VCA-IgA, VCA-IgG | 312 | 164 | 148 | - | - |
| Wu 2016 | China | Rta-IgG, VCA-IgA | 4084 | 170 | 3914 | 47 | 69.4% |
| Xia 2015 | China | EA-IgA, Rta-IgG, SA, VCA-IgA | 184 | 64 | 120 | 45.6 | 48.4% |
| Xiao 1995 | China | VCA-IgA | 5503 | 990 | 4513 | - | 70.2% |
| Xiao 2001 | China | EA-IgG | 120 | 60 | 60 | - | - |
| Yang 2006 | China | EBV-DNA plasma | 30 | 19 | 11 | - | - |
| Yang 2015 | China | EBV-DNA plasma | 270 | 220 | 50 | 52 | - |
| Yi 2007 | China | Zta-IgG | 52 | 24 | 28 | 56 | 77.8% |
| Yi 2018 | China | EA-IgA, Rta-IgG, VCA-IgA | 345 | 96 | 249 | 49.8 | 69.8% |
| Yip 2016 | China | EBV-DNA plasma | 107 | 48 | 59 | - | - |
| Yu 2016 | China | EBNA1-IgA, EBV-DNA plasma, VCA-IgA, Zta-IgA | 828 | 152 | 676 | - | 68.4% |
| Yuan 2002 | China | EBV-DNA plasma | 53 | 23 | 30 | 52 | - |
| Zeng 2010 | China | VCA-IgA | 1097 | 404 | 693 | - | - |
| Zhan 2009 | China | EA-IgA, VCA-IgA | 294 | 106 | 188 | - | - |
| Zhang 1998 | China | EA-IgG, VCA-IgA | 646 | 122 | 524 | - | - |
| Zhang 2002 | China | EA-IgA, EA-IgG, VCA-IgA | 609 | 264 | 345 | - | - |
| Zhang 2004a | China | EBV-DNA plasma, BV-DNA PMB | 222 | 146 | 76 | - | 74.7% |
| Zhang 2004b | China | EA-IgG, EBNA1-IgA | 112 | 54 | 58 | - | - |
| Zhang 2005 | China | SA | 150 | 50 | 100 | 51.1 | 54% |
| Zhang 2006 | China | Zta-IgG | 384 | 388 | 96 | - | - |
| Zhang 2015 | China | EA-IgA, Rta-IgG, VCA-IgA | 10587 | 156 | 10431 | 48 | 60.9% |
| Zheng 2009 | China | Rta-IgG | 624 | 211 | 413 | 48.4 | 73.9% |
| Zheng 2015 | China | EBV-DNA brushings, VCA-IgA | 162 | 128 | 34 | - | 74.2% |
| Zheng 2016 | China | EA-IgA, VCA-IgA | 424 | 215 | 209 | 47.1 | 73% |
| Zhou 1995 | China | VCA-IgA, EA-IgA | 200 | 50 | 150 | - | - |
| Zhou 2009 | China | EA-IgA, EA-IgG, VCA-IgA | 3561 | 145 | 3416 | 44.5 | 59.3% |
| Zhu 2008 | China | VCA-IgA | 95 | 62 | 33 | - | - |
| Zou 2001 | China | SA, VCA-IgA | 230 | 76 | 154 | - | - |

**Table S2.** PRISMA checklist.

| **Section/Topic** | **#** | **Checklist Item** | **Reported on page #** |
| --- | --- | --- | --- |
|  |  | **TITLE** |  |
| Title | 1 | Identify the report as a systematic review, meta-analysis, or both. | 1 |
|  |  | **ABSTRACT** |  |
| Structured summary | 2 | Provide a structured summary including, as applicable: background; objectives; data sources; study eligibility criteria, participants, and interventions; study appraisal and synthesis methods; results; limitations; conclusions and implications of key findings; systematic review registration number. | 1 |
|  |  | **INTRODUCTION** |  |
| Rationale | 3 | Describe the rationale for the review in the context of what is already known. | 1-2 |
| Objectives | 4 | Provide an explicit statement of questions being addressed with reference to participants, interventions, comparisons, outcomes, and study design (PICOS). | 1-2 |
|  |  | **METHODS** |  |
| Protocol and registration | 5 | Indicate if a review protocol exists, if and where it can be accessed (e.g., Web address), and, if available, provide registration information including registration number | 10 |
| Eligibility criteria | 6 | Specify study characteristics (e.g., PICOS, length of follow-up) and report characteristics (e.g., years considered, language, publication status) used as criteria for eligibility, giving rationale. | 10 |
| Information sources | 7 | Describe all information sources (e.g., databases with dates of coverage, contact with study authors to identify additional studies) in the search and date last searched. | 10 |
| Search | 8 | Present full electronic search strategy for at least one database, including any limits used, such that it could be repeated. | Method S1 |
| Study selection | 9 | State the process for selecting studies (i.e., screening, eligibility, included in systematic review, and, if applicable, included in the meta-analysis). | 10 |
| Data collection process | 10 | Describe method of data extraction from reports (e.g., piloted forms, independently, in duplicate) and any processes for obtaining and confirming data from investigators | 10 |
| Data items | 11 | List and define all variables for which data were sought (e.g., PICOS, funding sources) and any assumptions and simplifications made. | 10 |
| Risk of bias in individual studies | 12 | Describe methods used for assessing risk of bias of individual studies (including specification of whether this was done at the study or outcome level), and how this information is to be used in any data synthesis. | 11 |
| Summary measures | 13 | State the principal summary measures (e.g., risk ratio, difference in means). | 11 |
| Synthesis of results | 14 | Describe the methods of handling data and combining results of studies, if done, including measures of consistency (e.g., I2) for each meta-analysis. | 11 |
| Risk of bias across studies | 15 | Specify any assessment of risk of bias that may affect the cumulative evidence (e.g., publication bias, selective reporting within studies). | 11 |
| Additional analyses | 16 | Describe methods of additional analyses (e.g., sensitivity or subgroup analyses, meta-regression), if done, indicating which were pre-specified. | 11 |
|  |  | **RESULTS** |  |
| Study selection | 17 | Give numbers of studies screened, assessed for eligibility, and included in the review, with reasons for exclusions at each stage, ideally with a flow diagram. | 2+Figure 1 |
| Study characteristics | 18 | For each study, present characteristics for which data were extracted (e.g., study size, PICOS, follow-up period) and provide the citations. | 2-3+Table S1 |
| Risk of bias within studies | 19 | Present data on risk of bias of each study and, if available, any outcome level assessment (see item 12). | 7+Table 2+ Figure 3+ Figure S1-14 |
| Results of individual studies | 20 | For all outcomes considered (benefits or harms), present, for each study: (a) simple summary data for each intervention group (b) effect estimates and confidence intervals, ideally with a forest plot. | 3-7+Table1+ Figure 2 |
| Synthesis of results | 21 | Present results of each meta-analysis done, including confidence intervals and measures of consistency. | 3-7+Table1 |
| Risk of bias across studies | 22 | Present results of any assessment of risk of bias across studies (see Item 15). | 7+ Table 2+ Figure 3+ Figure S1-14 |
| Additional analysis | 23 | Give results of additional analyses, if done (e.g., sensitivity or subgroup analyses, meta-regression [see Item 16]). | 7+ Table 2 |
|  |  | **DISCUSSION** |  |
| Summary of evidence | 24 | Summarize the main findings including the strength of evidence for each main outcome; consider their relevance to key groups (e.g., healthcare providers, users, and policy makers). | 8+10 |
| Limitations | 25 | Discuss limitations at study and outcome level (e.g., risk of bias), and at review-level (e.g., incomplete retrieval of identified research, reporting bias). | 9-10 |
| Conclusions | 26 | Provide a general interpretation of the results in the context of other evidence, and implications for future research. | 9-10 |
|  |  | **FUNDING** |  |
| Funding | 27 | Describe sources of funding for the systematic review and other support (e.g., supply of data); role of funders for the systematic review. | 13 |
